# Microbiota-derived short chain fatty acids in fermented Kidachi Aloe promote antimicrobial, anticancer, and immunomodulatory activities

**DOI:** 10.1186/s12866-023-02981-z

**Published:** 2023-08-29

**Authors:** Lamiaa A. Al-Madboly, Akira Yagi, Amal Kabbash, Mona A. El-Aasr, Rasha M. El-Morsi

**Affiliations:** 1 Department of Microbiology and Immunology, Faculty of Pharmacy, Tanta University, Tanta, Egypt; 2 Department of Pharmaceutical Science, Fukuyama University, Hiroshima, Japan; 3 Department of Pharmacognosy, Faculty of Pharmacy, Tanta University, Tanta, Egypt; 4 Department of Microbiology, Faculty of Pharmacy, Delta University for Science and Technology, International Coastal Road, Gamasa, 11152 Egypt

**Keywords:** Microbiota, Biological activities, Fermented juice, *A. arborescens*

## Abstract

**Background:**

Fermented Aloe leaf juice is a commonly used food supplement in Japan. In a previous study, fermentation of *A. arborescence* juice was performed and the presence of short-chain fatty acids (SCFAs) was confirmed and quantified. Samples were collected before and after the fermentation process to be subjected, in the present study, to DNA extraction, 16S rRNA gene (V3–V4 regions) amplification, and sequencing by the next-generation Illumina MiSeq sequencer. Our work aims to analyze the sequences to assess the bacterial diversity in the juice before and after fermentation, identify the beneficial microbes responsible for the production of SCFAs, and evaluate some of the biological activities of the fermented juice.

**Results:**

Data revealed the richness and diversity of the bacterial community in the fermented juice compared to the unfermented control. Relative abundance of bacterial phyla showed that the majority of the microbial community in the test samples corresponded to *Pseudomonadota* (unfermented; 10.4%, fermented; 76.36%), followed by Bacillota (unfermented; 4.71%, fermented; 17.13%) and then *Bacteroidota* (unfermented; 0.57%, fermented; 1.64%). For the fermented sample, 84% of Bacillota were lactobacilli. A hierarchically clustered heatmap revealed that *Lactobacillus* was the most abundant genus in both samples suggesting its involvement in the production of SCFAs.

To assess potential health benefits, the anticancer efficacy of the fermented product of *A. arborescens* was investigated against colorectal cancer (IC50 = 3.5 µg/ml) and liver cancer (IC50 = 6.367 µg/ml) compared to the normal peripheral blood mononuclear cells (PBMCs). Flow cytometric analysis of the cell cycle pattern revealed remarkable population arrest in G0 and G1, however, the highest percentages were mainly in the G1 phase for Hep-G2 (40.1%) and HCT-116 (53.2%) cell lines. This effect was accompanied by early apoptotic profiles of HCT-116 (36.9%) and late apoptosis for Hep-G2 (17.3%). Furthermore, immunomodulatory properties demonstrated a significantly (*p* < 0.001) reduced percentage of induced TNF-α while enhancing IFN-γ dramatically. For antimicrobial activities, marked broad-spectrum activities were recorded against some bacterial and fungal pathogens (17–37 mm inhibition zone diameter range).

**Conclusion:**

Therefore, this study affords the basis of bacterial community composition in fermented *A. arborescens* juice as well as its potential biological benefits.

**Supplementary Information:**

The online version contains supplementary material available at 10.1186/s12866-023-02981-z.

## Introduction

Different Aloe species are commonly used in traditional medicine because of their therapeutic properties. Two species are considered the most important; *Aloe vera* as well as *Aloe arborescens* Mill. [1]. In particular, *A. arborescens* which is native to the mountainous regions of Southern Africa was first introduced to Japan in the seventeenth century where it became naturalized [2]. It is characterized by morphologically variable species, specifically, *A. arborescens* var. *natalensis* (known as Kidachi Aloe in Japan) as concluded by Reynolds [3] who regarded it as *A. arborescens* because of being a variable species rather than attempt to give varietal names (*A*. *arborescens* var. *natalensis*) for the overseas growth forms. The inner leaf gel of *A*. *arborescens* is enriched with typical polysaccharides including; aloerides, acemannans, and pectins which exhibit useful therapeutic properties such as anti-bacterial, anti-inflammatory, wound-healing, antioxidant, and immune-stimulant [4]. Furthermore, phenolic metabolites such as anthraquinones as well as their glycosides derivatives (barbaloin, aloin A, and aloin B) are present in very low content, hence *A. arborescens* does not have laxative effects [5].

Fermented foods have been used by humans for a long history due to being associated with a lot of health benefits such as improvements in blood pressure, cholesterol levels, reduction in gut irregularity, and low risk of type II diabetes [6, 7]. To produce products of fermented food, live microorganisms can be transferred to food. Alternatively, fermentation can be carried out depending on natural endophytic bacteria commonly associated with plants such as those of Aloe species leading to modification in the physical as well as nutritional characteristics through breaking down complex compounds forming useful by-products [8]. Due to the important role of endophytic bacteria in fermented food, massive sequencing technologies are used together with bioinformatics to analyze the bacterial diversity and their function during or after the fermentation process providing information, particularly, about the uncultured bacterial communities in such ecosystems [9, 10].

It is worth mentioning that there are no previous studies about the microbiota and their role in fermented *A. arborescens* juice. The literature only provides information about the applications of fresh juice as an antifungal against the trichosporon genus and yeasts, antiviral activity against some respiratory viruses, and the chemopreventive effect of a dried whole leaf [10–12]. Additionally, *A. arborescens* juice was also reported to have anti-skin wrinkling effects after the addition of *Lactiplantibacillus plantarum* and subjection to fermentation. It effectively promoted collagen production and inhibited MMP-1 synthesis in human fibroblast cells [13]. A Toxicological study, performed to evaluate the toxicity of barbaloin administered in rats, revealed increased incidences of intestinal goblet cell hyperplasia in a dose-related manner. Additionally, this effect was extended from the cecum to the rectum when a dose of 55.7 mg/kg was given to rats [14]. Additionally, it could also reduce butyrate production by fecal microbiota 24 h after administration of high doses [15]. Consequently, trials to reduce the barbaloin content of *A. arborescens* juice by fermentation may enhance its safe therapeutic use. Interestingly, the present work is considered the first report about the metagenomic analysis of microbiota diversity as well as the biological activities of the fermented juice of kidachi Aloe leaf.

## Materials and methods

### Amplification of target DNA fragment, Illumina MiSeq sequencing, and analysis

Leaves of *A. arborescens* were collected from the herbal garden of Fukuyama university. Details related to sample collection and fermentation conditions of *A. arborescens* juice were previously reported by Kabbash et al. [16]. Briefly, fermentation was done in the presence of 20% W/W sucrose for one month at room temperature. Three samples from three fermentation batches were tested. Before the start of fermentation, three samples were also picked to represent the unfermented control. Both fermented and unfermented samples were subjected to extraction of total genomic DNA using a DNeasy PowerSoil Kit (QIAGEN, DE), as per the manufacturer’s instructions. The concentration and quality of DNA were checked at A_260/280_ nm (1.9–2.0) using a Nanodrop Spectrophotometer. The 16S rRNA gene (V3-V4 conserved regions) was amplified using the primers 341F as well as 805R (Macrogen, Inc., Korea) with a 12 nt unique barcode; forward primer (5ʹ-TCGTCGGCAGCGTCAGATG TGTATAAGAGACAGCCTACGGGNGGCWGCAG-3ʹ) and reverse primer (5ʹ-GTCTCGTGGGCTCGGAGATGTGTATAAGAGACAGGACTACHVGGGTATCTAA TCC-3ʹ) [17]. Briefly, each PCR reaction of 25 μL contains 10 ng/μL (6 μL) of template genomic DNA, about 12.5 μL 2 × Mastermix (1 U), 1.5 μL of each primer (10 μM), and finally nuclease-free water. PCR reactions condition was adjusted to be as follows; initial denaturation step at 95°C for 3 min, then followed by 24 cycles where the temperature was adjusted at 98°C for 20 s, 55 °C for 15 s, and 72°C for 10 s and terminated with an extension step at 72°C for 1 min. Triplicates of PCR reaction were carried out, and the amplicons were confirmed using agarose gel electrophoresis. Samples showing a positive electrophoresis band (550bp) were selected and OMEGA Gel Extraction Kit (Omega Bio-Tek, USA) was used to purify this band, and DNA was quantified. PCR amplicons were sent to the IGA Technology sequencing company (Italy) for 16 s rRNA gene sequencing [18]. Samples were subjected to purification using Agencourt XP Ampure Beads (Beckman Coulter, USA). For library preparation, the Nextera XT index kit (Illumina, USA) was used. Assessment of library quality was done by Bioanalyzer 2100 (Agilent Technologies, USA) and the product quantification was carried out by Qubit (Invitrogen, Thermofisher, USA). Samples were passed in equal proportion into the Illumina MiSeq sequencer (Illumina, USA) and subjected to paired-end sequencing with about 600 cycles (12 cycles for barcode and 300 cycles for each paired-end read sequence).

### Processing of the sequences

Forward and reverse reads of raw data were merged using SeqPrep 1.2 related to the EBI pipeline alignment method (pipeline software; http://www.ebi.ac.uk/metagenomics).

### Data analysis and microbial community composition

Data was imported into QIIME2 in Casava 1.8 demultiplexed paired-end format (2019.7). To analyze data, this was begun by the generation of an interactive sequence quality plot so nonbiological sequences including; primers, sequencing adapters, and PCR spacers were filtered out using DADA2 plugin, which also could correct errors in the marginal sequences, remove noisy and chimeric sequences, as well as singletons.

QIIME2 feature-classifier was used to extract reads from silva-138–99 database into ASV table with a 99% identity threshold.

Alpha diversity richness was assessed using QIIME2 by a variety of metrics such as Shannon–Wiener index, OTUs_observed, and Chao1. MGnify was used to request analysis following data archive in ENA. Furthermore, InterProScan annotations and GO terms predicted the functional content of microbial communities based on the sequencing data using MGnify [19]. A heatmap presenting data was prepared by reducing the sample set of 30 OTUs for simplification of the matrix. So, the heatmap was generated to present the relative abundance of taxa in both fermented and unfermented samples where Bray Curtis similarity was used for clustering.

### Nucleotide sequence accession number

The sequence of 16S rRNA genes hypervariable region was deposited in the European Nucleotide Archive (ENA) of the EBI database under the accession number PRJEB51954, ERP136620, and MGnify ID MGYS00005311.

### Assessment of fermented juice biological activities

#### Mammalian cell lines

Human colorectal carcinoma (HCT-116) and hepatocellular carcinoma (HepG-2) were purchased from the tissue culture department of the Holding Company for the Production of Vaccines, Sera, and Drugs (VACSERA-EgyVac). Normal non-cancerous cells; human peripheral blood mononuclear cells (PBMCs) were cultured on RPMI-1640 or MEM media supplemented with 200 mM L-glutamine and 10% fetal bovine serum. Peripheral blood mononuclear cells (PBMCs) were isolated by gradient centrifugation, as reported by Verreck et al. [20].

### Cell proliferation inhibition assay

Fermented *A. arborescens* juice was subjected to sterile filtration and the filtrate was freeze-dried then a stock solution (10 µg/mL) was prepared in media. For the assessment of the dose-dependent cell proliferation inhibition effect of the test product, HepG-2 or HCT-116 cells were propagated in 75 cm^2^ cell culture flasks using RPMI-1640 medium supplemented with 10% (v/v) fetal bovine serum and 1% penicillin–streptomycin (Invitrogen, USA) and then incubated in 5% CO_2_ incubator at 37 °C for 24 h. Confluent cells were detached using 0.25% (w/v) trypsin solution and 0.05% (v/v) ethylenediaminetetraacetic acid (EDTA) (Gibco-USA) for 5 min. Cells were plated at a concentration of 2 × 10^5^ cells/ml in 96-well cell culture plates and incubated at 37 °C for 24 h to achieve confluency. The medium was decanted and a fresh medium containing serial dilutions of the test product was added for cytotoxicity determination using colorimetric 3-(4,5-dimethyl-2-thiazolyl)-2,5-diphenyl-2 H-tetrazolium bromide (MTT) reduction assay. Dead cells were washed out using PBS, and 50 μl of MTT stock solution (5 mg/ml) was added to each well. After a 4 h incubation period, the supernatants were discarded and the formazan precipitates were solubilized by the addition of 50 μl / well of dimethyl sulfoxide (DMSO). Plates were incubated in the dark for 30 min at a temperature of 37 °C, and absorbance was determined at a wavelength of 570 nm using a microplate reader. The cell viability percentage was calculated and the assay was performed three times. The cell viability (%) was plotted against the tested Aloe concentrations. The IC_50_ value of the tested product was determined using the Masterplex-2010 software program. The experiment was performed in triplicate. Additionally, the anticancer activities of fermented *A. arborescens* juice against cancer cells were determined by monitoring the morphological alterations of the treated cells using an inverted microscope [21].

### Cell cycle analysis

The alterations in cell cycle pattern were determined using flow cytometry according to [22]**.** In this assay, propidium iodide (PI) can be employed to discriminate living cells from dead cells, and for cell cycle analysis that is based on the stoichiometric binding of PI to intracellular DNA. Hep-G2 cells precultured in 25 cm^2^ cell culture flasks were treated with an IC_50_ of the test product dissolved in RPMI-1640 medium, for 24 h. Cells were then washed with warm PBS and collected by trypsinization. For cell cycle analyses, the collected cells (about 2 × 10^5^ cells/mL) were then resuspended in warm PBS, fixed gently with about 4 ml ice-cold 70% (v/v) ethanol, maintained at a temperature of 4 °C overnight, and then stained with 0.5 mL of warm PI solution (7 mL of the PI solution consists of 0.35 mL of PI solution (1 mg/mL), 0.7 mL RNase A solution (1 mg/mL), 6 mL of PBS and 0.1% (v/v) Triton X-100) in a dark room. After 30 min of incubation at 37 °C, cells were then analyzed using a flow-cytometer (Becton–Dickinson, San Jose, CA, USA) equipped with an argon-ion laser at a wavelength of 488 nm. The obtained cell cycle profile and sub-G_1_ group were analyzed using CELL QUEST version 3.2 and Win MDI version 2.8 software [23]. The experiment was carried out three times. Flow cytometric analysis was performed at the Center of Excellence and Cancer Research (CECR), Tanta University, Egypt.

### Detection of apoptosis using annexin V staining and propidium iodide (PI)

The appearance of phosphatidylserine (PS) residues (normally hidden within the plasma membrane) on the surface of the cell is an early event in apoptosis and can be used to detect and measure apoptosis. During apoptosis, PS is translocated from the cytoplasmic face of the plasma membrane to the cell surface. Annexin V is a Ca^2+^-dependent phospholipid-binding protein with a high affinity for PS. Hence this protein can be used as a sensitive probe for PS exposure upon the cell membrane. Annexin V binding was assessed using bivariate FCM according to Martin et al. [24], and cell staining was evaluated with fluorescein isothiocyanate (FITC)-labeled Annexin V (green fluorescence), simultaneously with dye exclusion of propidium iodide (PI) (negative for red fluorescence). Hep-G2 or HCC-116 cancer cells were placed and seeded into a 6-well culture plate treated with the IC50 of the fermented juice, incubated for 24 h then subjected to the quantitative determination of apoptotic cells. Quadrant statistic calculations were done using CELLQUEST PRO software (BD Bioscience, San Jose, CA). The experiment was repeated three times with triplicate samples for each experiment [25].

### Immunomodulatory activities of *A. arborescens* fermented juice

Quantification of the tumor necrosis factor-alpha (TNF-α) and interferon-gamma (IFN-γ) in LPS‑induced PBMC cells; the immunomodulatory role of test product on human PBMCs was investigated in vitro. Its effect on the expression of TNF-α and IFN-γ in the LPS-induced PBMC model was studied using flow cytometric analysis (intracellular staining protocol). About 2 × 10^6^ cells/mL of PBMCs were suspended in RPMI medium and seeded into a rounded bottom, 6-well plate for 24 h. At the end of incubation, the inflammatory model was induced by stimulating PBMC with 100 μl of *E. coli* LPS (20 ng/ml) for 24 h in the presence or absence of the treatment (IC_50_ of fermented Aloe juice). The intracellularly induced cytokines were assessed by flow cytometric analysis using FITC-labeled anti-human TNF-α and FITC-labelled anti-human IFN-γ, respectively. The experiment was repeated thrice with triplicate samples for each experiment. The flow cytometric analysis was performed at the Center of Excellence and Cancer Research (CECR), Tanta University, Egypt [26].

### Evaluation of the antimicrobial effects of the fermented juice

#### Effect on bacterial and fungal pathogens

Mueller–Hinton or Sabouraud Dextrose Agar was seeded with appropriate well-mixed overnight nutrient broth culture of each test microorganism to 0.5 McFarland turbidity standard: *Staphylococcus aureus* (ATCC 25923), *Bacillus cereus* (ATCC 14579), *Escherichia coli* (ATCC 26922), *Salmonella* *typhi* (ATCC 6539), *Shigella flexneri* (ATCC 12022), *Helicobacter pylori* (ATCC 43504), *Listeria monocytogenes* (ATCC 19115), *Vibrio cholera Incertae sedis* (ATCC 14035), and *Candida albicans* (ATCC 90028). About 1 × 10^6^ colony forming units (CFU) per milliliter were used, by spreading evenly onto the surface of the medium. Wells were cut from the agar plates using a sterile cork borer [27]. The wells were loaded with the test juice (50 µg/mL) allowing a 10 min diffusion time. The plates were incubated at 37 °C or 28 °C for fungi for 24 h thereafter the plates were examined for any clearing zones around the wells. The zones of inhibition were measured in mm. This experiment was repeated three times for confirmation. Diameter means and standard deviations were calculated. For determination of the minimum inhibitory concentration, it was performed according to the EUCAST [28].

### Statistical analysis

Data were statistically analyzed using Statistical Package for Social Science version 20 (SPSS; Armonk, NY: IBM Corp.). The student’s unpaired t-test was used for pairwise comparisons analysis whereas the one-way analysis of variance (ANOVA) test was used for comparison among different groups followed by Bonferroni post hoc test. Each experiment was repeated at least three times and data are shown as mean ± SE and *p* < 0.05* is considered statistically significant while *p* < 0.001** is highly significant.

## Results

### Bacterial community composition in fermented and unfermented samples of *A. arborescence* juice

**Fig. 1:**
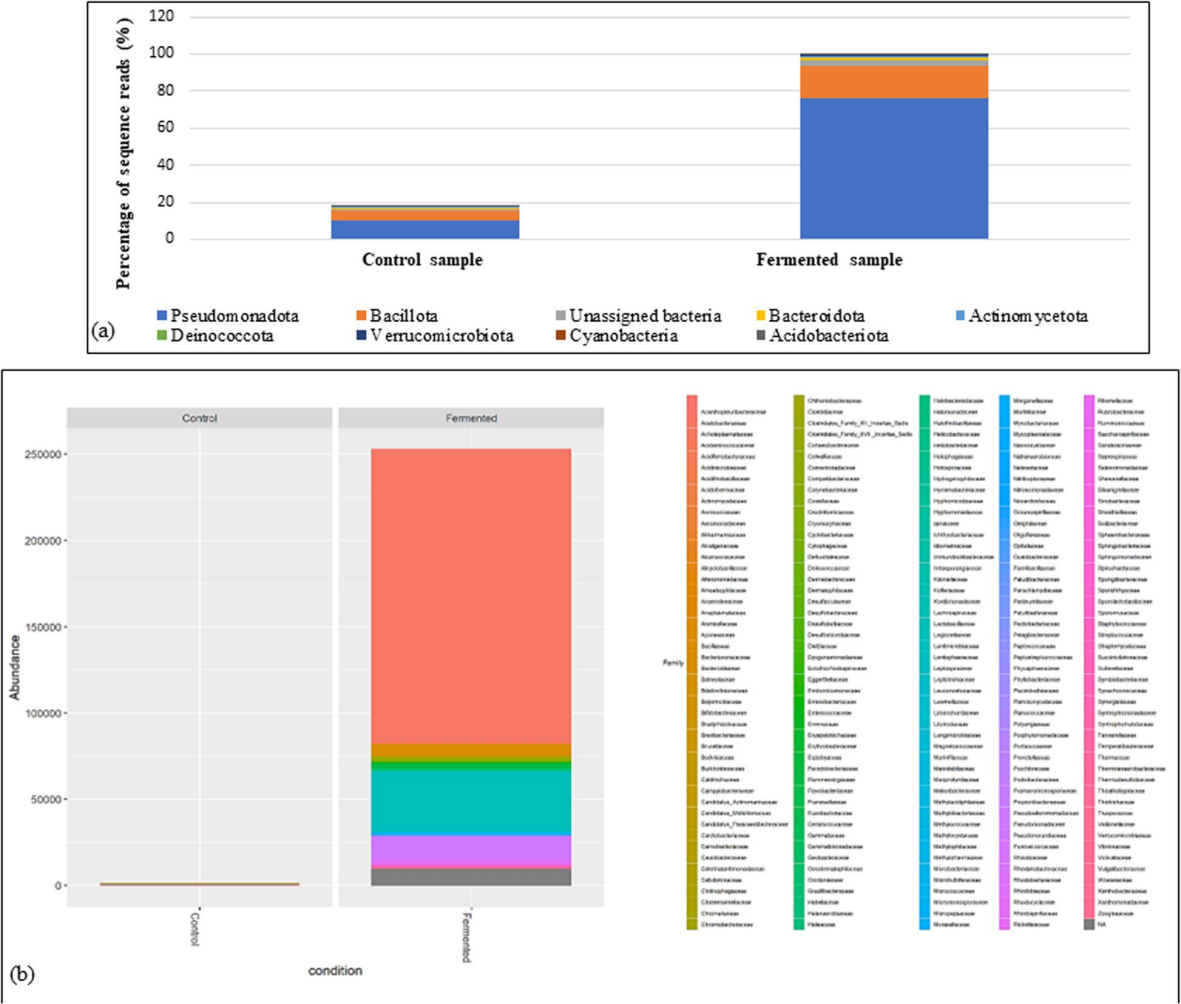
Abundance of the bacterial community in both fermented and unfermented samples of *A. arborescens* juice showing the richness of fermented juice with high diversity **a** at the phylum level, and **b** at the family level

Analysis of richness (observed_OTUs), singletons (Chao1 value), and equity (Shannon index) which indicate the alpha-diversity within bacterial communities, revealed very high significant differences between fermented and unfermented samples (Table 1 and Fig. 1, a and b). The bacterial community in the fermented product was highly diverse recording the highest values in tested diversity indices compared to the unfermented control (Table 1 and Fig. 1, a and b).

**Table 1:** Alpha diversity values

Sample	Alpha diversity metrics		
	**Observed-OTUs**	**Chao1 index**	**Shannon index**
**Fermented sample**	710.300 ± 6.410^*^	1000.11 ± 5.758^*^	2.27 ± 0.091^*^
**Unfermented sample**	21.571 ± 3.652	10.224 ± 2.450	2.09 ± 0.015

^*^High significant difference with *p* < 0.001

**Fig. 2:**
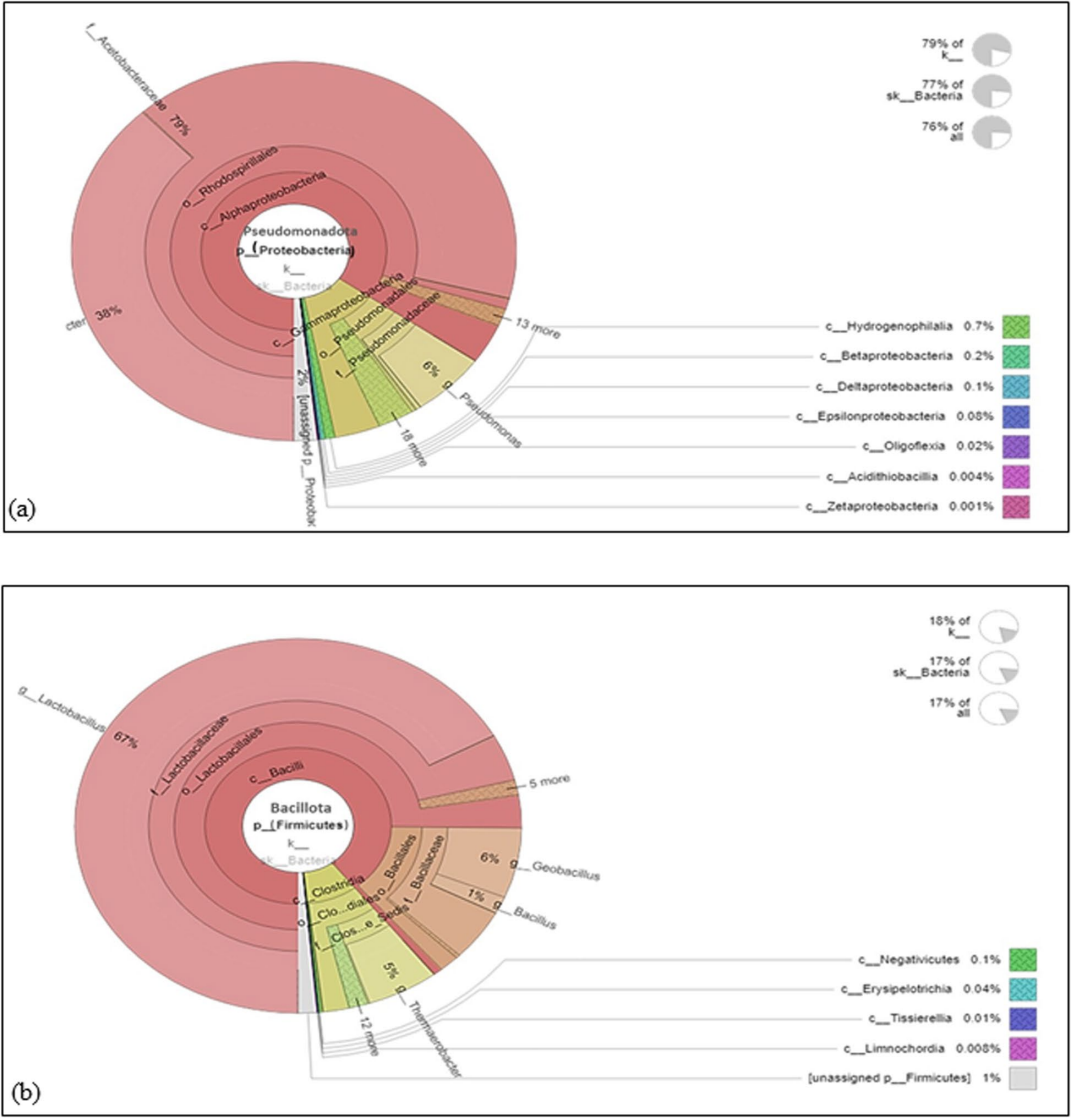
Taxonomic analysis of data related to fermented sample showing that *Acetobacteriaceae* represent the majority of *Pseudomonadota* (formerly; Proteobacteria) while *Lactobacillus* is the main genus related to *Bacillota* (formerly; Firmicutes)

The relative abundance of bacterial phyla was assessed using MGnify showing that the majority of the microbial communities in the test samples corresponded to *Pseudomonadota* (unfermented; 10.4%, fermented; 76.36%), followed by Bacillota (unfermented; 4.71%, fermented; 17.13%) and then Bacteroidota (unfermented; 0.57%, fermented; 1.64%) as displayed in Fig. 1a. For the fermented sample, the majority of bacterial composition (65%) belonged to *Pseudomonadota* as shown in Fig. 2a. However, 84% of Bacillota were lactobacilli and only 9% were Clostridia (Fig. 2b).

A phylogenetic tree at the family level was generated for fermented sample data and presented in the supplementary Fig. S1. Members of family *Acetobacteraceae* recorded the most assignments (79%) followed by *Lactobacillaceae* (12%), *Clostridiales* family XVII Incertae Sedis (1%), and *Flavobacteriaceae* (1%). Moreover, three important bacterial families were also detected including; *Lachnospiraceae* (0.7%), *Ruminococcaceae* (0.2%), and *Clostridiales* XII Incertae Sedis (0.2%) as presented in supplementary Fig. S2a.

For family *Acetobacteraceae*, two important organic acid-producing genera were detected in the fermented sample including; *Acetobacter* (38%) which was the most common one as well as *Komagataeibacter* (0.1%) as presented in the supplementary Fig. S2b. Among the Firmicutes, genus *Lactobacillus* recorded 67% of family *Lactobacillaceae*. Other important and beneficial genera were also detected including; *Thermobacter* (98% of *Clostridiales* XVII), *Ruminococcus* (98% of *Ruminococcaceae*)*, Faecalibacterium* (1% of *Ruminococcaceae*), *Anaerostipes* (6% of *Lachnospiraceae*), *Roseburia* (6% of *Lachnospiraceae*), *Streptococcus* (0.5%) and *Enterococcus* (1%) as shown in supplementary Figs. S2b, S3). Two phylogenetic trees were generated at the genus level for both proteobacteria and firmicutes of the fermented sample as presented in supplementary Figs. S4 and S5. Interestingly, a neighbor-joining phylogenetic diversity of Firmicutes sequences at the order level was generated (supplementary Fig. S6) showing Lactobacillales presented as a major order as indicated by a relatively large-sized circle.

**Fig. 3:**
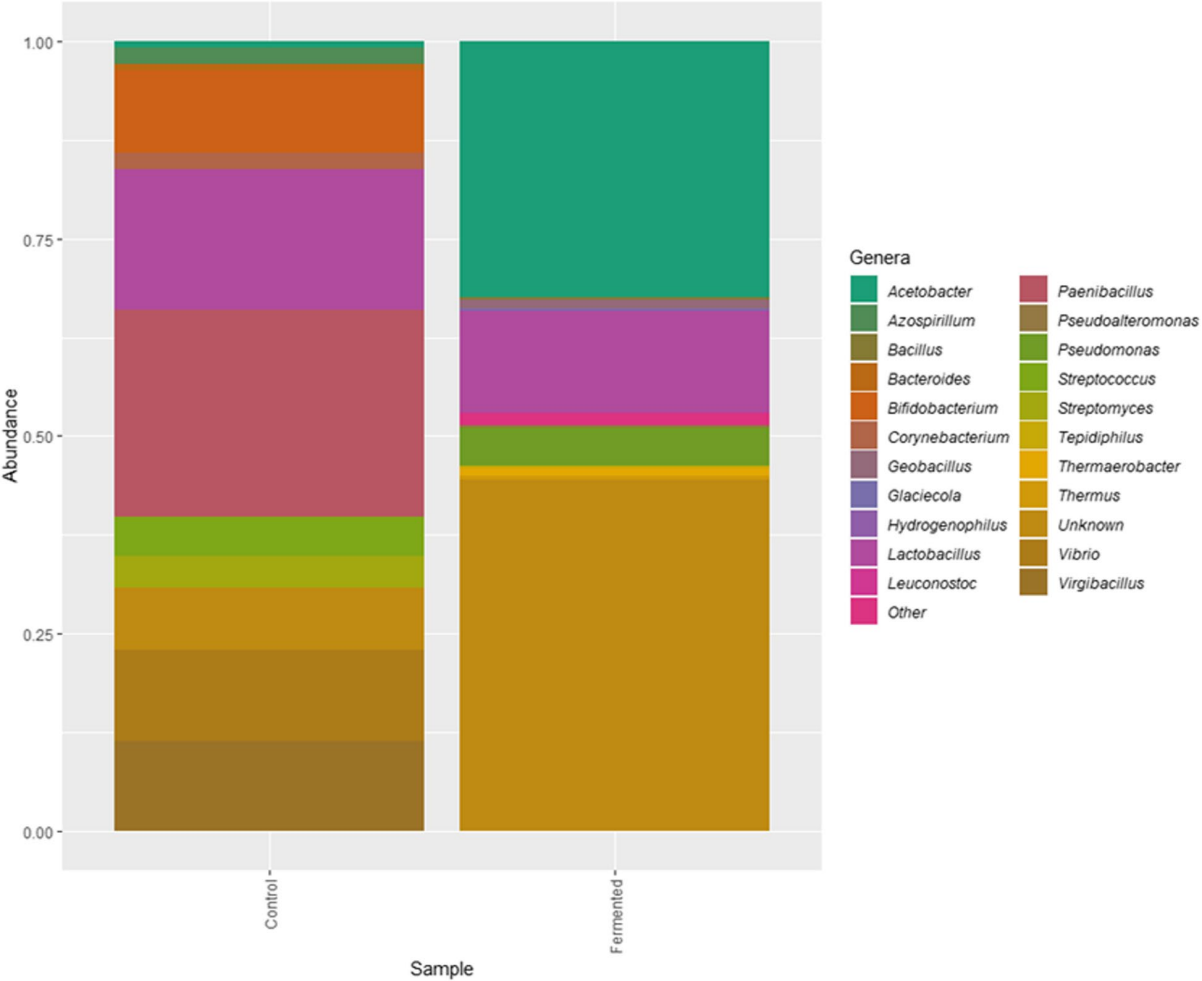
Relative abundance percentage of bacterial genera detected in the fermented as well as the un-fermented samples of *A. arborescence* juice

Figure 3 presented the bacterial genera detected in both fermented as well as unfermented samples and their relative abundance. The most commonly detected, was the genus *Lactobacillus*.

**Fig. 4:**
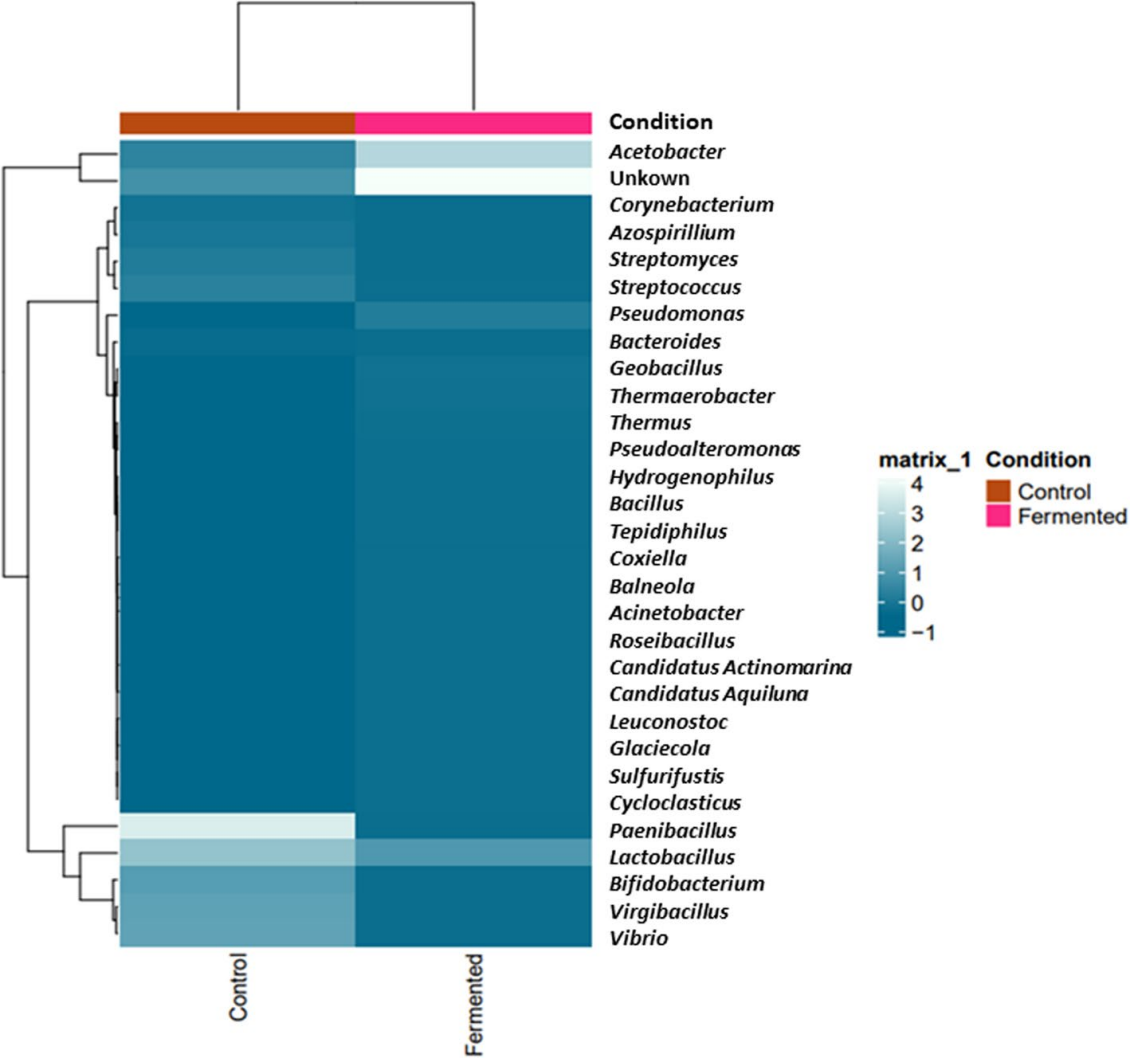
Hierarchically clustered heatmap presenting bacterial genera distribution in both fermented as well as unfermented samples of *A. arborescence* juice. Each column represents a sample and each row stands for the relative abundance of each bacterial genus, which showed by the color intensity with the legend designated at the right of the figure

To get an overall assessment of the identified bacterial genera between fermented and unfermented samples, a hierarchically clustered heatmap was generated (Fig. 4). The plot of the heatmap depicted the relative percentage of each bacterial genus (variables clustering on the X-axis) within each sample (Y-axis clustering). For each bacterial genus, the relative abundance was represented by the color intensity in the legend shown at the right of the figure. It was found that *Lactobacillus* was the most abundant genus in both samples.

**Fig. 5:**
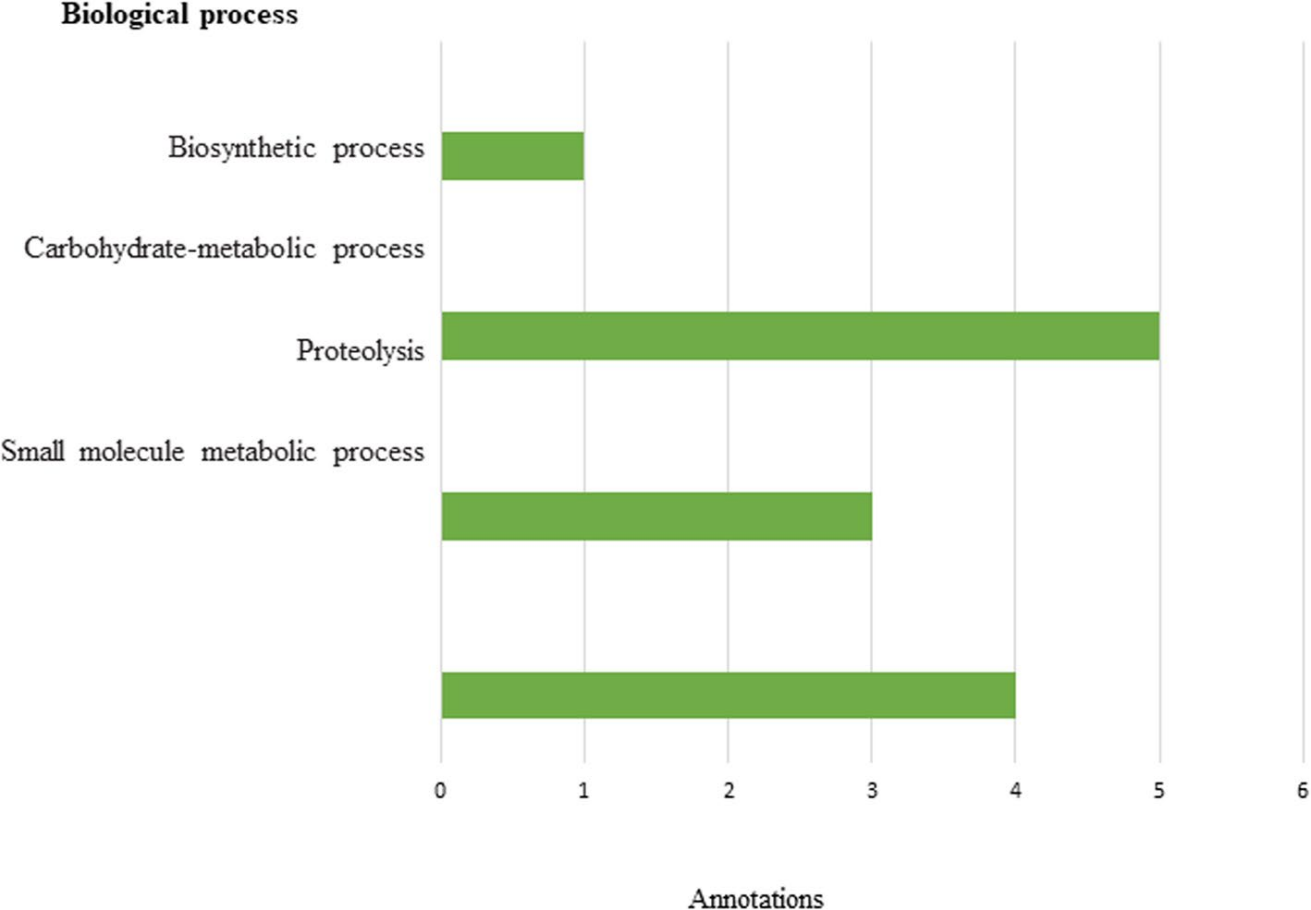
Annotations of biological processes predicted by MGnify

**Fig. 6:**
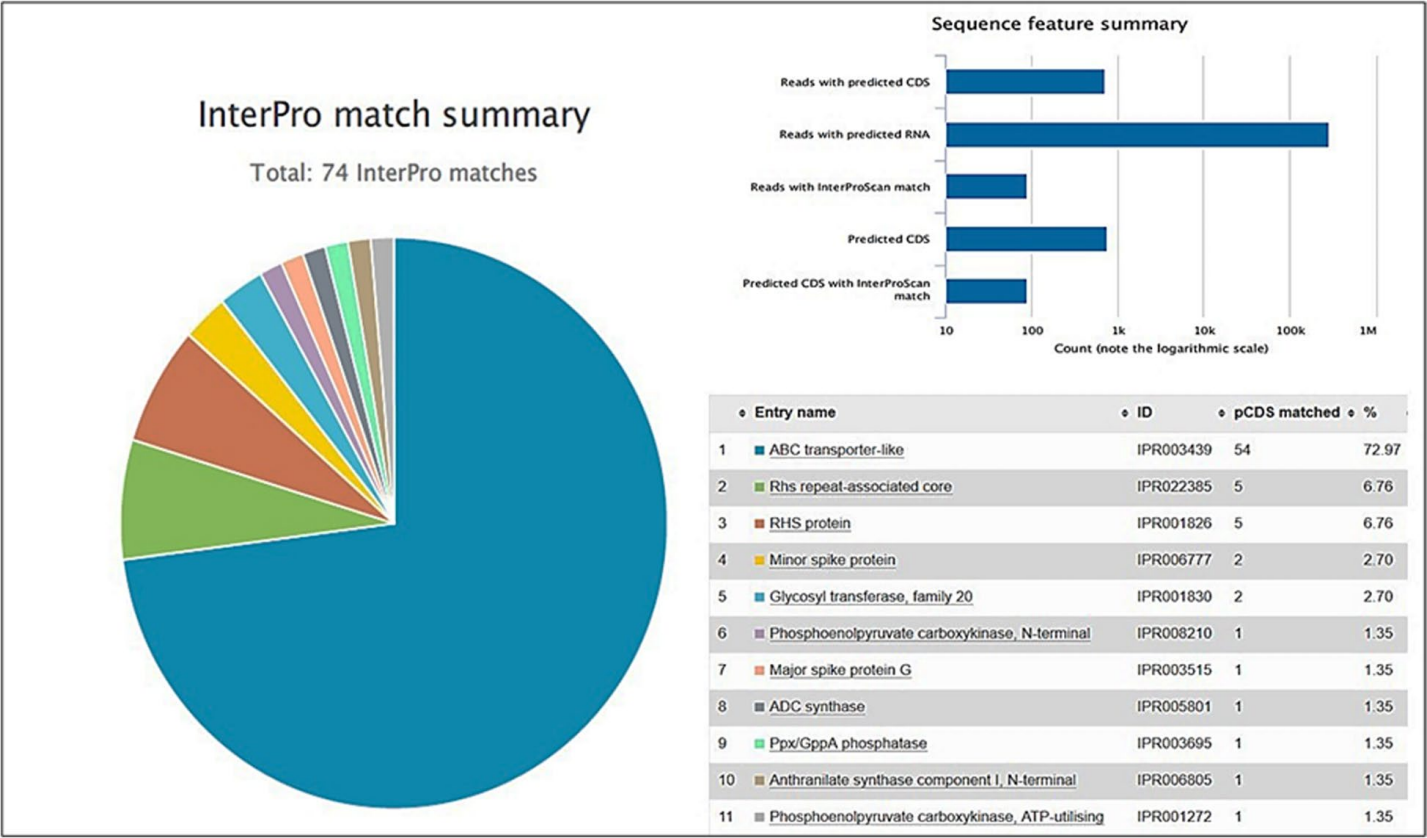
Functional analysis of EBI pipeline depending on the matches to Interpro and Go-terms showing top 11-matches and ABC transporter-like recorded the highest matching

Gene function annotation of the bacterial community was predicted by MGnify and presented in Figs. 5 and 6. The major functional profiles of bacterial genes were categorized into carbohydrate transport and metabolism, biosynthetic process, proteolysis (amino acid metabolism), and small molecule metabolic process (Fig. 5). InterPro match database classified protein into 11 families and predicted the presence of catalytic domains as shown in Fig. 6. For example, ABC transporter-like recorded 72.97% pCDS matching as shown in Fig. 6.

### Evaluation of different biological activities of Fermented juice of *A. arborescence*

#### Cytotoxicity assay of *Aloe arborescens* juice

**Fig. 7:**
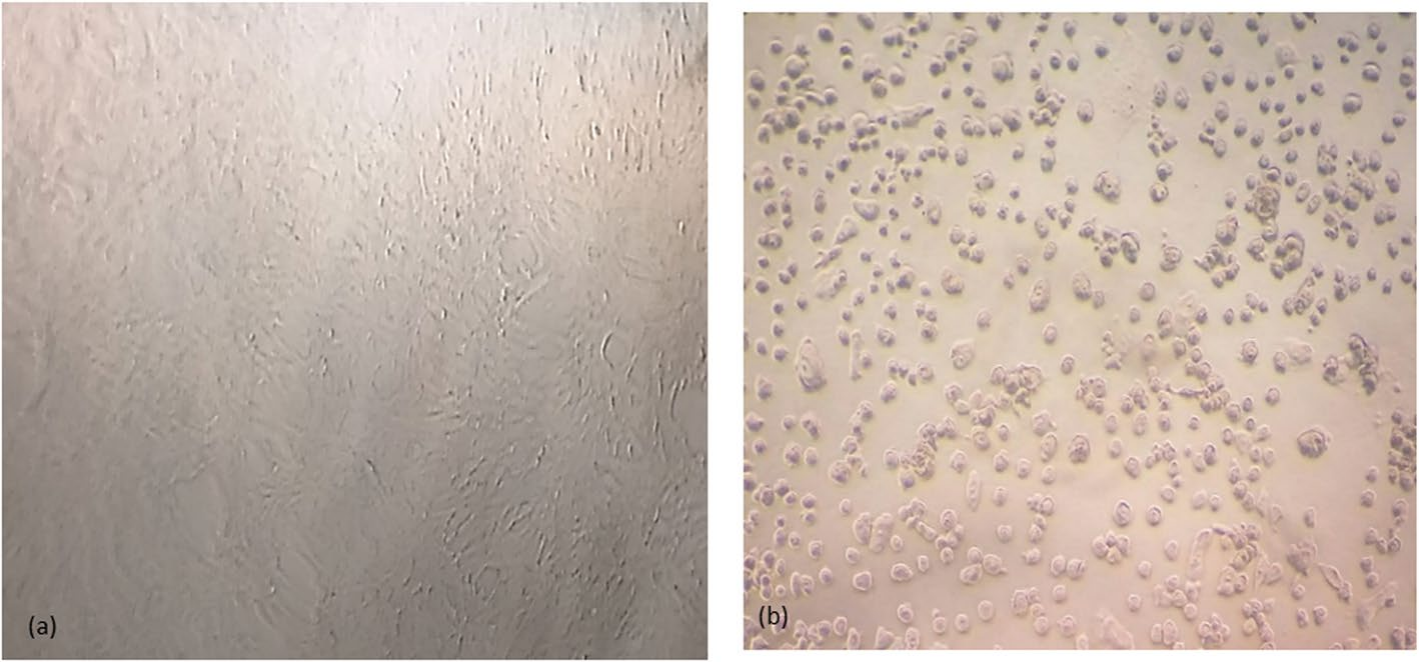
Cytotoxicity assay of fermented *A. arborescence* juice on **a** Hep-G2, **b** HCT-116, and **c** normal PBMCs using MTT assay

**Fig. 8:**
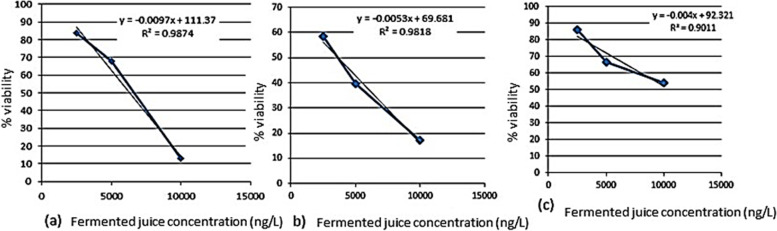
Cytotoxic effect of fermented *A. arborescence* juice showing the morphological changes of HCT-116 cells 24 h post-treatment using an inverted microscope: **a** Untreated (control) cells showing polygonal shape with homogenous cellular contents. **b** Treated cells showing membrane blebbing, cellular deformation with severe shrinkage, and condensation of cell contents

The cytotoxic effect of the fermented Aloe on Hep-G2, HCT-116 colon cancer cells, and PBMCs was assessed using MTT assay. The antiproliferative potential of the fermented Aloe showed concentration-dependent cell viability, it was noticed that cell viability was concentration-dependent as the cytotoxicity was significantly increased at higher concentrations of the tested juice (Fig. 7). The half-maximal inhibitory concentration (IC_50_) of Aloe juice was relatively low against HCT-116 cell line (3.5 µg/ml) and Hep-G2 cell line (6.367 µg/ml) compared to the normal PBMCs (11 µg/ml). Various morphological abnormalities of the cells before and after treatment were also recorded. The untreated culture fields showed a polygonal shape with distinct boundaries and homogenous cellular contents (Fig. 8a). On the contrary, induced cell toxicity was associated with morphological changes 24 h post-treatment with the fermented Aloe juice. Figure 8b showed the appearance of undergoing apoptotic cells that are characterized by cellular rounding up, deformation with severe shrinkage and condensation of their cellular contents, membrane blebbing, cells detached out of the culture surface, and loss of cell adhesion. Data related to unfermented juice were presented in the supplementary Fig. S7 where the situation is reversed as the safe concentration on PBMCs is low concentration (IC50 = 3.7 µg/ml) while the cytotoxic concentration against cancer cell lines is relatively high (Hep-G2; IC50 = 7.8 µg/ml, and HCT-116; IC50 = 6.5 µg/ml).

### Cell Cycle analysis and apoptosis

**Fig. 9:**
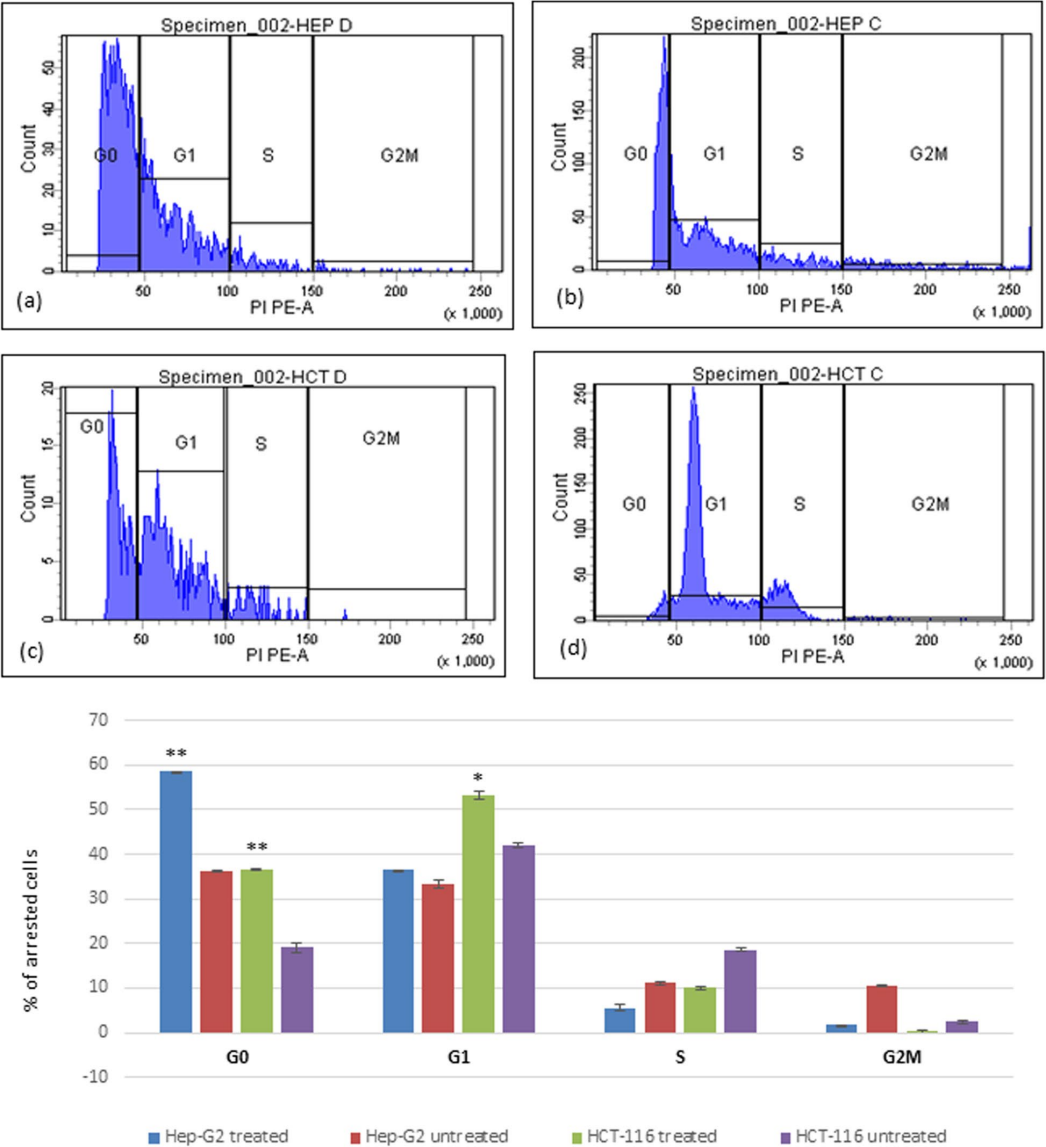
The effect of fermented Aloe juice treatment on the regulation of the cell cycle of colon and liver cancer cells, **a** untreated Hep-G2 cells, **b** treated Hep-G2 cells, **c** untreated HCT-116 cells, and **d** treated HCT-116 cells. The percentage and distribution of arrested cells in the pre-phase (G0), G1, S, and G2/M phases of the cell cycle are shown in the graph presented. (*P < 0.05, **P < 0.001, Student’s unpaired t-test)

**Fig. 10:**
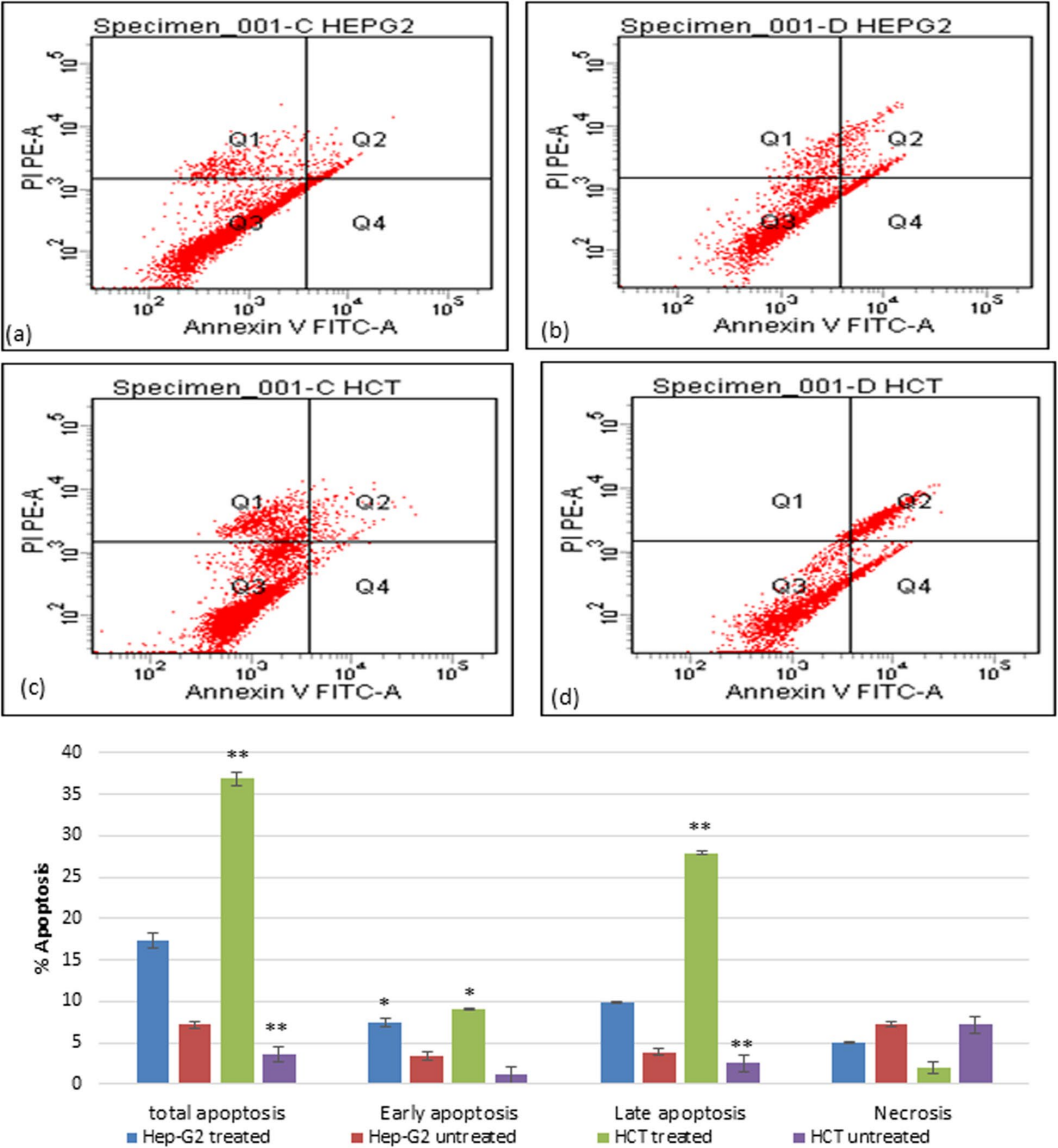
Flow cytometric analysis of untreated Hep-G2 cells (**a**), treated-Hep-G2 liver cancer cells (**b**), untreated HCT-116 (**c**), and treated-HCT-116 colon cancer cells (**d**). Dots with Annexin V-/PI- (Q3), Annexin V+/PI- (Q4), and Annexin V+/PI+ (Q2) Annexin V-/PI+ (Q1) features represent viable intact, early apoptotic, late apoptotic cells and necrotic cells, respectively. The percentages of arrested cells in each apoptosis phase, as well as necrosis, are indicated in the presented graph. (**P <0.001, Student’s unpaired t-test)

The flow cytometric analysis for the cell cycle pattern of the treated cells was shown in Fig. 9. After treatment, the population percentages in the G0 and G1 that represented the apoptotic cells were increased significantly (*p* < 0.001** & 0.05*, respectively) in HCT-116 treated cells to reach 36.4 & 53.2%, respectively in comparison to the untreated control cells. A similar effect was recorded for treated Hep-G2 cells where 36.9 and 40.1% of the population were arrested significantly (*p* < 0.001**) in G0 and G1, respectively. Additionally, it was noticed that cell arrest was accompanied by early and late apoptotic profiles recording a total of 36.9 and 17.3% (*p* < 0.001**) apoptotic cells of HCT-116 and Hep-G2, respectively. The highest percentage was in the late apoptotic phase (27.9 & 9.9%, respectively, *p* < 0.001**) as presented in Fig. 10. Necrosis was also observed (2.2 and 5%, *p*˂ 0.05*) in treated HCT-116 and Hep-G2, respectively.

### Immunomodulatory activities of fermented *A. arborescens* juice

**Fig. 11:**
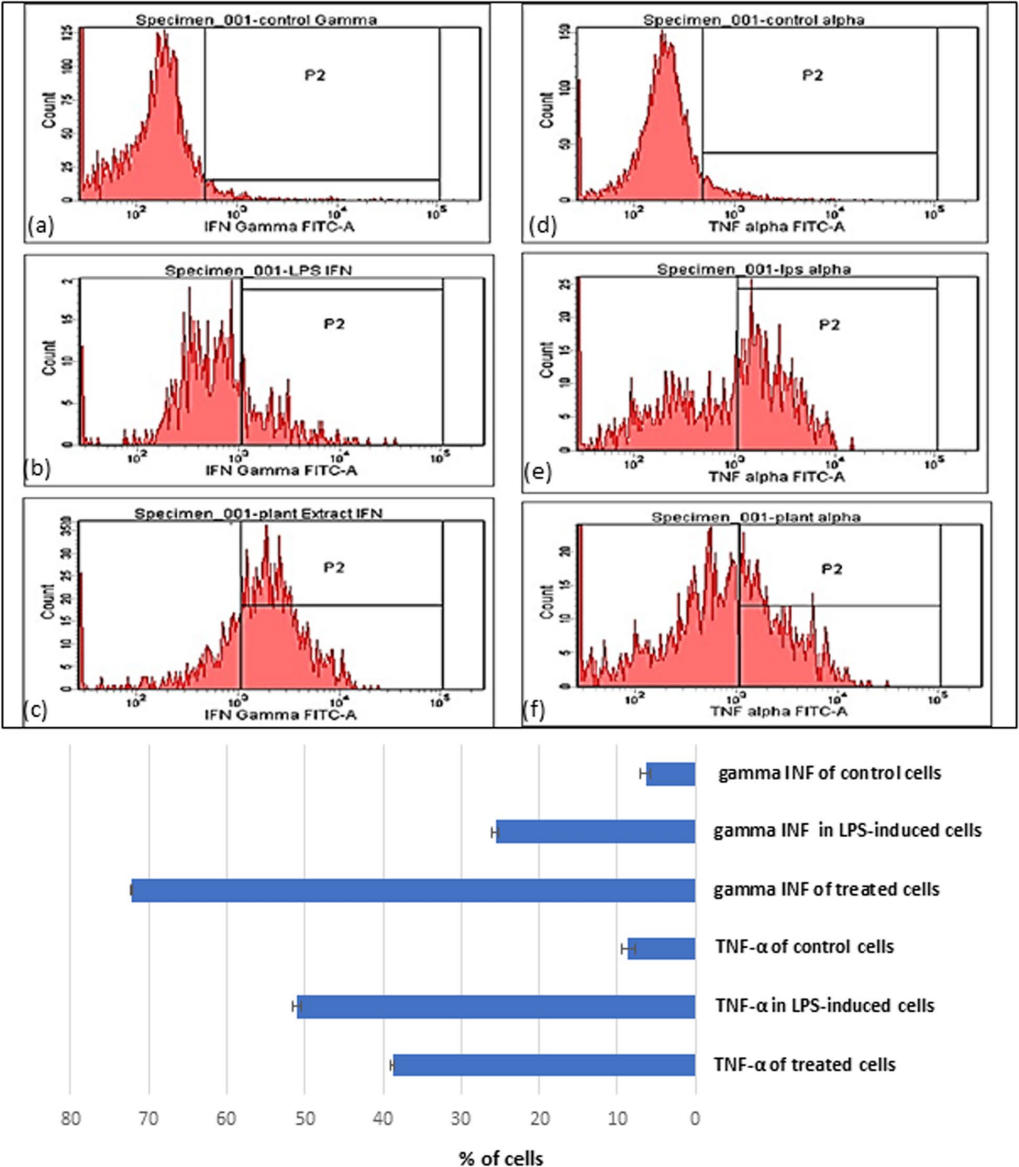
Flow cytometric analysis showing the effects of fermented Aloe juice on the TNF-α and IFN-γ production by LPS-induced PBMC cell model. Cells were incubated with or without LPS (1mg/mL) for 24 h, in the presence or absence of Aloe at the indicated concentration. **a** untreated PBMCs negative control for INF-γ, **b** LPS-induced PBMCs (Positive control for INF-γ), **c** Aloe-treated PBMCs for INF-γ, **d** untreated PBMCs negative control for TNF-α, **e** LPS-induced PBMCs (Positive control for TNF-α) and **f** Aloe-treated PBMCs for TNF-α. Data are presented in the graph as mean ± SE where statistical significance was tested using one-way ANOVA (*P < 0.05, ** *P* < 0.001)

Quantification of the tumor necrosis factor-alpha (TNF-α) and interferon-gamma (IFN-γ) in the LPS‑induced PBMC cell model: At least 2 × 10^4^ cells were analyzed using flow cytometry after treatment with the non-toxic concentration of the fermented Aloe juice for 48 h. The induced intracellular TNF-α and IFN-γ levels in the PBMCs cell model after treatments were quantified by measuring the emitted relative fluorescence of the fluorescent probe using flow cytometry. TNF-α was detected in approximately 51.1% of LPS‑induced PBMC cells from healthy donors but this percentage was reduced up to 38.8% after treatment. On the other hand, IFN- γ was detected in 25.6% of LPS‑induced PBMC cells from healthy donors while detected in 72.2% of the cells following treatment. Thus, according to the obtained results, it was found that fermented aloe juice significantly (*p* < 0.001**) reduced the percentage of induced TNF-α while enhancing IFN-γ dramatically as shown in Fig. 11. Data related to the effect of unfermented juice on the immunomodulatory properties were presented in the supplementary fig. S8 where a slight nonsignificant decrease (49.4%; *p* > 0.05) in the percentage of TNF-α producing cells was detected however, IFN-γ was recorded in 32.7% of LPS‑induced PBMC cells (*p* > 0.05).

### Evaluation of the antimicrobial effect of fermented *A. arborescens* juice

#### Effect on bacterial and fungal pathogens

**Fig. 12:**
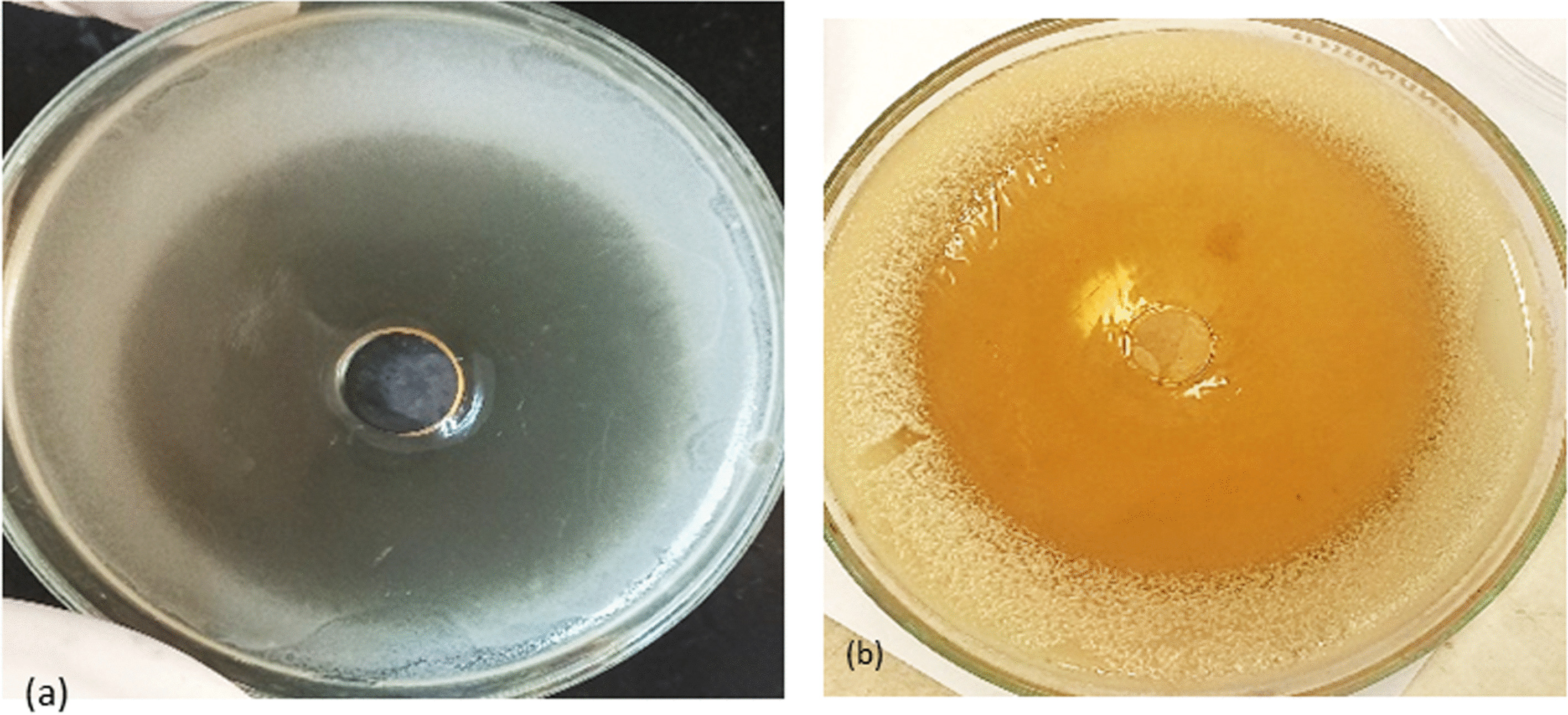
Antimicrobial effect of fermented *A. arborescens* product against *E. coli* **a**, and *C. albicans* **b**, showing large inhibition zone diameters

The fermented Aloe juice displayed broad-spectrum antibacterial, as well as antifungal activities recording inhibition zone diameters ranging between 17- 37 mm (Fig. 12, Table 2). Statistical analysis of data using ANOVA revealed significant differences between different groups (*p* < 0.05) and Bonferroni's Multiple Comparison Test showed significant differences (*p* < 0.05) between the effect of fermented juice against Gram-positive and the other pathogens. Furthermore, MIC values recorded against most tested bacteria were relatively lower than that recorded against *C. albicans* as shown in Table 2. Assessment of antimicrobial activities of unfermented juice was performed and data presented in supplementary Table S1.

**Table 2:** Evaluation of antimicrobial activities of *A. arborescens* fermented juice using the agar well-diffusion method and MIC determination by broth microdilution assay

**Test pathogens**	**Inhibition zone diameters** (mm)^a^	**MIC** (µg/mL)
***S. aureus***	37 ± 1	4
***B. cereus***	31 ± 0.57	4
***E. coli***	21 ± 1	8
***S.*** *typhi*	19 ± 0.57	8
***Sh. flexneri***	19 ± 1.15	8
***H. pylori***	18 ± 1.15	2
***L. monocytogenes***	19 ± 1	4
***V. cholera***	17 ± 0.57	4
***C. albicans***	20 ± 0.57	8

^a^Values were presented as mean inhibition zone (mm) ± SD of triplicates

## Discussion

In previous work, we prepared the fermented extract of *A. arborescens* leaves juice and determined the fermentation products using HPLC and GC/MSD [16, 29]. Data revealed relatively high concentrations of short-chain fatty acids (SCFAs) particularly butyric acids (0.15%), acetic acid (0.024%), and propionic acid (0.01%) while a low amount of the major phenolic compound barbaloin (14 μg/mL) was recorded, compared to the unfermented control. Additionally, lactic acid (19.12%) was also recorded in the fermented sample. In the present work, we were interested in determining the bacterial diversity in *A. arborescens* fermented juice and identifying the taxa which were responsible for the relatively high concentrations of SCFAs (lactic acid and butyric acid) and the reduced amount of barbaloin. Another aim of this study was to evaluate some of the biological effects of the fermented extract.

The interaction of endophytic bacteria with *A. arborescens* leads to a compromise between both mutualism and antagonism to generate a harmonious symbiotic system. *A. arborescens* fermented extract displayed a possible promotion of host plant growth to get a balanced living environment [30]. In the present study, bacterial sequencing data revealed *Pseudomonadota*, *Bacillota*, *Actinomycetota*, and *Bacteriodota* as dominant phyla in the fermented Aloe juice. The highest percentage was recorded for *Pseudomonadota* (78.36%) followed by *Bacillota *(17.58%) indicating abundance. Based on the analyzed bacterial population of the fermented juice and gene prediction function, *Lactobacillus* was the most common genus to which we might attribute the production of SCFAs. Akinsanya et al. [31] reported similar results about the predominant bacteria in *Aloe vera*.

The production of butyrate had gotten much attention due to its anti-neoplastic and anti-inflammatory effects on colonocytes [32, 33]. Butyric acid promotes mucin synthesis and tightens the junctions existing between the epithelial cells, hence preventing inflammation as well as a leaky gut syndrome. That is why butyrate-producing bacteria shield the human gut against inflammation, colorectal cancer, and ulcerative colitis [34]. Butyrate can be synthesized directly from carbohydrate sources as reported by Kabbash et al. [16] who stated that the fermentation of *A. arborescens* leaf juice was done in the presence of 20% W/W sucrose for one month at room temperature. Additionally, butyrate might be produced indirectly through bacterial bioconversion because there is a relationship between gut microbiota and the production of SCFAs that was reported in the literature. Butyrate can be synthesized from acetic or lactic bioconversion by butyrogenic bacteria [34, 35]. Interestingly, *Lactobacillus* was predominant in both fermented and unfermented samples of *A. arborescens* as confirmed by the heatmap. This genus is commonly attributed to the production of lactic acid which is detected in a relatively high concentration in the fermented juice and could be converted into butyrate in the human gut [16, 36, 37]. Additionally, *Acetobacter* was also detected and it is known to be an important source of acetic acid production. This genus also could inhibit the growth of food poisoning-triggering pathogenic bacteria via secreting certain by-products and hence possess the properties of probiotics [38–40]. Moreover, Bacteroides could also produce SCFAs from lactic acid bioconversion and they could digest fats and whole grains [41]. They were also detected in the fermented sample in a small percentage (1.64%) relative to the firmicutes (17.13%) because if their quantity exceeds, they might be harmful and so, it is important to detect firmicutes in higher ratios than Bacteroides, up to 2–3 times [34]. Accordingly, it is important to mention that endophytic bacteria present in the juice of *A. arborescens* leave are responsible for SCFAs production such as propionic, acetic, butyric, and lactic acid in relatively high concentrations through in vitro fermentation, and are capable of bioconversion within the human gut. This was also supported by the data of the predicted gene function annotation of the bacterial community in the fermented juice that revealed carbohydrate metabolism as a major functional profile.

A second aim of the present study is to evaluate some of the health-promoting attributes of the fermented product including; anticancer efficacy, immunomodulatory, and antimicrobial effect. So, its safety against normal human PBMCs was assessed first using MTT assay, recording an IC_50_ of 11 µg/ml. Interestingly, we reported in our previous work [16] that the barbaloin content of *A. arborescens* fermented product was dramatically reduced from 600 ppm (in fresh tissue) to 14 ppm (by endophytic bacteria) following the fermentation process. Moreover, the later concentration could be diluted to less than 10 ppm if it is used as an Aloe beverage or soft drink and hence it is safe according to the International Aloe Science Council (IASC) standard. Ro et al. [13] reported that investigating a concentration range of 0.01- 0.5% of *A. arborescens* extract fermented by *Lactiplantibacillus plantarum* did not show any cellular cytotoxicity against human fibroblast cells denoting safety. Moreover, the enhanced mitochondrial activity of normal human fibroblasts was also stated. For anticancer efficacy of the fermented product of *A. arborescens*, it was investigated in the current study against colorectal cancer (HCT-116) as well as liver cancer cell line (HepG2). Results of the MTT assay revealed very low IC_50_ against HCT-116 (3.5 µg/ml) and Hep-G2 (6.367 µg/ml) compared to the normal PBMCs. Shalabi et al. [42] reported that the IC_50_ value of *A. vera* extract was (10.45) μg/ml against HepG2. Nazeam et al. [43] mentioned that water-soluble polysaccharides and acid-soluble polysaccharides extracted from *A. arborescens* leave exerted significant cytotoxicity against HepG2 cells, with IC_50_ values of 26.14 and 21.46 μg/mL, respectively. This variation in IC_50_ values is due to the differences in methods used in Aloe preparation.

In cancer chemotherapy, it is preferable for a drug to induce apoptosis to inhibit cell growth and proliferation, so, we tested the ability of the fermented product to induce cancer cell suicide [44]. On the morphological level, treated cancer cells showed cellular apoptotic features; cell shrinkage, chromatin condensation, plasma membrane blebbing, and apoptotic bodies. Flow cytometric analysis of the cell cycle pattern revealed remarkable population arrest in G0 and G1. Moreover, the highest percentages were mainly in the G1 phase (40.1 and 53.2) for Hep-G2 and HCT-116, respectively. Additionally, it was noticed that cell arrest was accompanied by early apoptotic profiles of HCT-116 (36.9%) while late apoptosis for Hep-G2 (17.3%). Our results were in agreement with Shalabi et al. [42] who mentioned that *A. vera* extract triggered apoptosis through up-regulation of *P53* and down-regulation of *Bcl-2* gene expressions. Anwar et al. [45] reported similar results where *A. arborescens* extract could inhibit colorectal tumorigenesis in an animal model.

Immunomodulatory activities of the fermented product were also assessed in the present study via flow cytometric quantitation of TNF-α and IFN-γ. The test product showed a significant reduction in the percentage of induced TNF-α while exerting a dramatic increase in the IFN-γ level. The former cytokine known to be pro-inflammatory is involved in many inflammatory conditions and can also promote tumor development [46], and hence its reduction means an anti-inflammatory effect of *A. arborescens* fermented product. For IFN-γ, it has a dynamic role in triggering a range of immune responses [47]. Sensitized T-cells and natural killer (NK) primarily secrete IFN- γ to mediate antitumor as well as antiviral immunity, activate antigen-presenting cells, promote the innate immune system, control Th1/Th2 balance, and regulate the cellular proliferation as well as apoptosis [48]. Additionally, Anwar et al. [45] reported that* A. arborescens* extract increased the production of IFN-γ in an animal model with induced liver cancer.

Regarding the antimicrobial activities of *A. arborescens* fermented product, our results showed marked broad-spectrum antimicrobial activities displaying large inhibition zone diameters against some important food-borne bacterial pathogens as well as opportunistic pathogens like *C. albicans*. Similarly, Stanley et al. [49] stated that *A. vera* gel showed inhibitory effects against pathogenic bacteria, causing different human diseases, particularly *S. aureus* and *E. coli.* On the other hand, Kupnik et al. [50] reported that *C. albicans* was less susceptible when exposed to *A. arborescens* juice. However, *E. coli* and *P. aeruginosa* recorded MIC values of 200 and 600 µg/ml, respectively.

In conclusion, our study determined bacterial community composition and evaluated the anticancer, antimicrobial, and immunomodulatory activities of *A. arborecens* fermented product. Data analysis revealed bacterial diversity enriched with organic acid-producing microorganisms placing the groundwork for the vast potential of health benefits, particularly the anticancer effect where we investigated the direct antitumor effects on colorectal cancer and liver cancer cell lines, as well as, the immunomodulatory activities. This study revealed that our product exerted a direct cytotoxic action on the tumor cells via apoptotic mechanisms in addition to stimulating the immune response and providing novel targets to the current therapeutic manipulation of cancer. Moreover, Aloe fermented product showed a broad-spectrum activity as antimicrobial. Finally, further comparative studies are required to understand discrepancies in microbial diversity, fermentation time, and brewing process that may affect the quality of the fermented product and its impact on health.

### Supplementary Information

**Additional file 1.**

## Data Availability

The raw data supporting the conclusions of this article will be made available by the authors, without undue reservation, to any qualified researcher. The datasets generated and/or analysed during the current study are available in the the European Nucleotide Archive (ENA) of the EBI database repositoryt under the accession number PRJEB51954, ERP136620.
