# Supplementary material for: Microbiota-derived short chain fatty acids in fermented Kidachi Aloe promote antimicrobial, anticancer, and immunomodulatory activities
Source: BMC Microbiol. 2023 Aug 29;23:240. doi: 10.1186/s12866-023-02981-z (PMC10464184; doi:10.1186/s12866-023-02981-z)
Supplement: Supplementary file 1 — Additional file 1. [file 12866_2023_2981_MOESM1_ESM.docx]

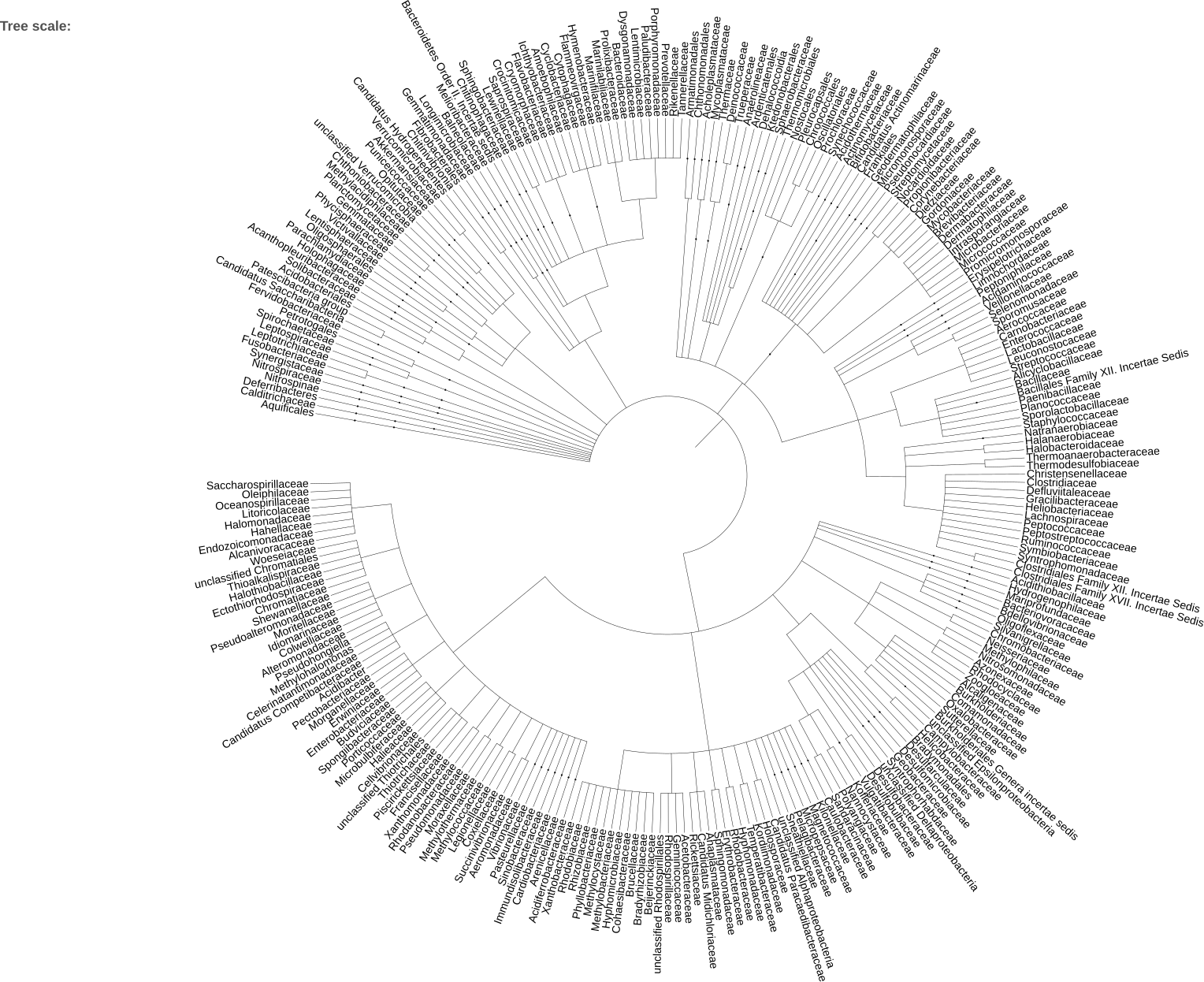


**Figure (S1):** Circular phylogenetic tree showing various bacterial families in the fermented juice sample. Arrows refer to the most important families.


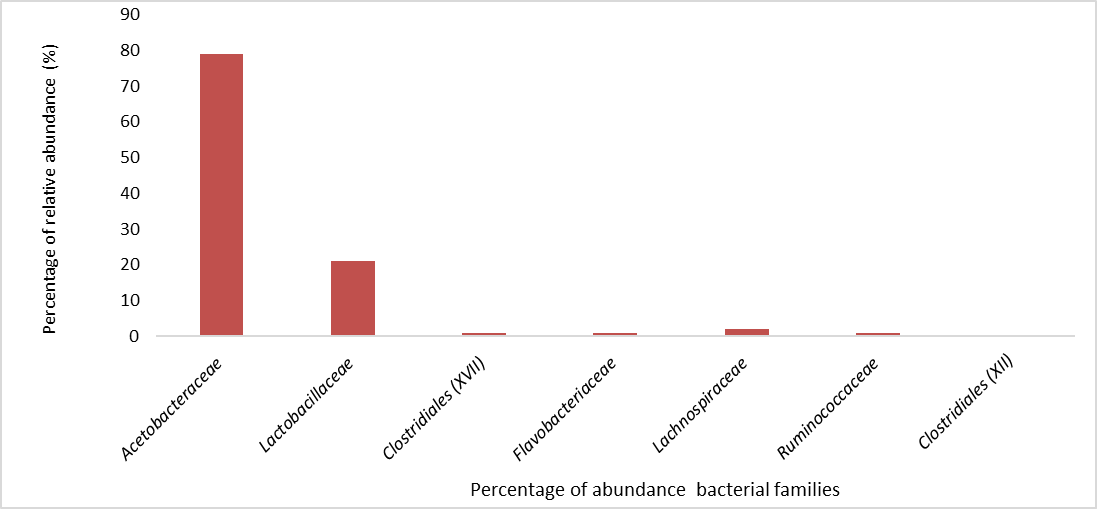


(a)


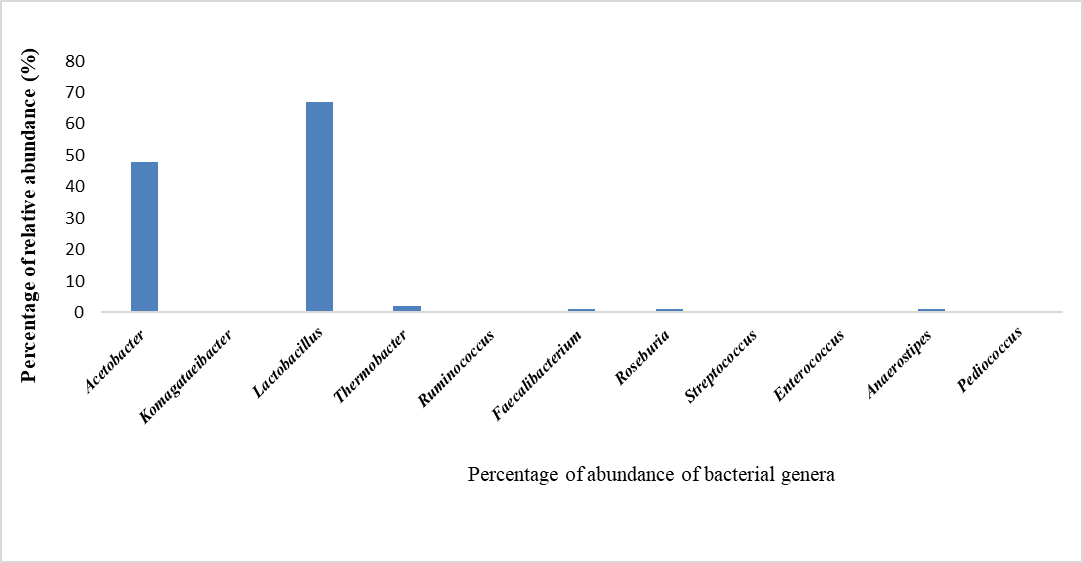


(b)

**Fig. (S2):** Relative abundance of the most common and most important bacterial (a) families, (b) genera, that detected in the fermented juice of *A. arborescens.*

**Figure (S2):** Relative abundance of the most common and most important bacterial (a) families, (b) genera, that detected in the fermented product of *A. arborescens.*


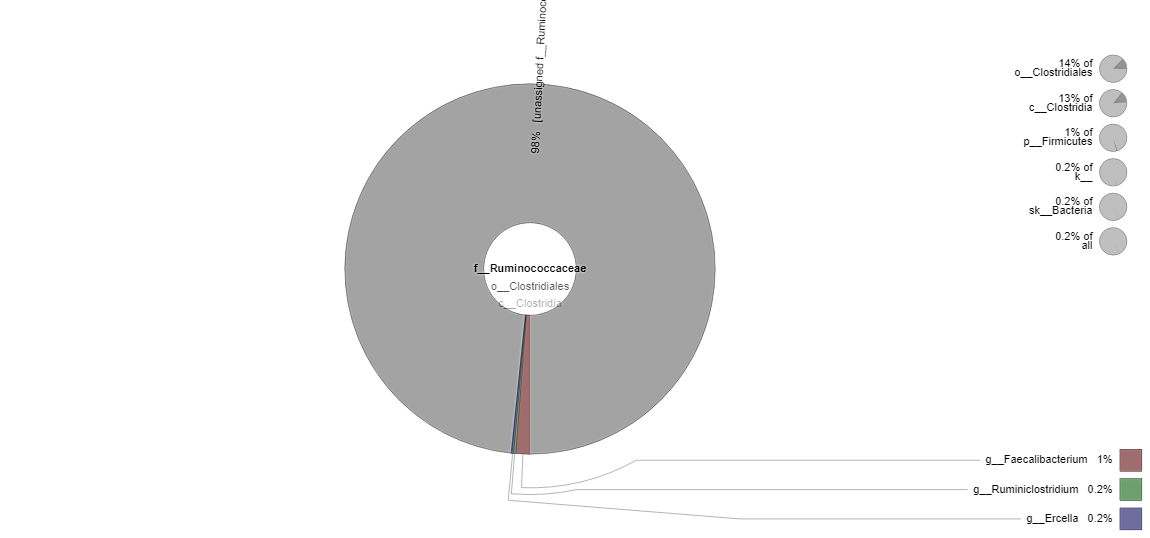


(a)


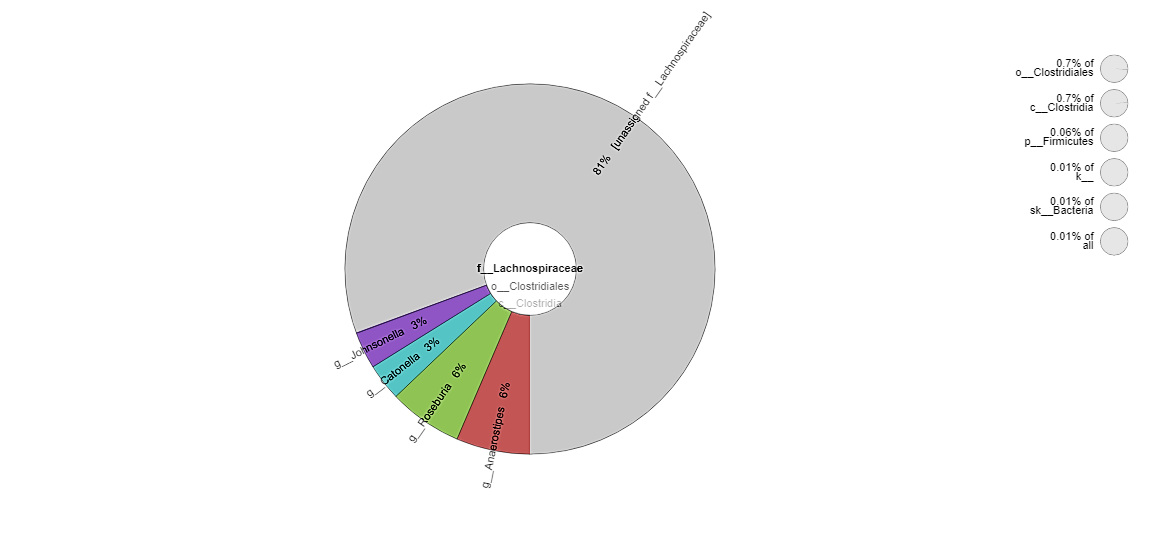


(b)

**Figure (S3):** Taxonomic analysis of data related to fermented sample showing abundance percentage of (a) *Faecalibacterium*, (b) *Roseburia* and *Anerostipes*.


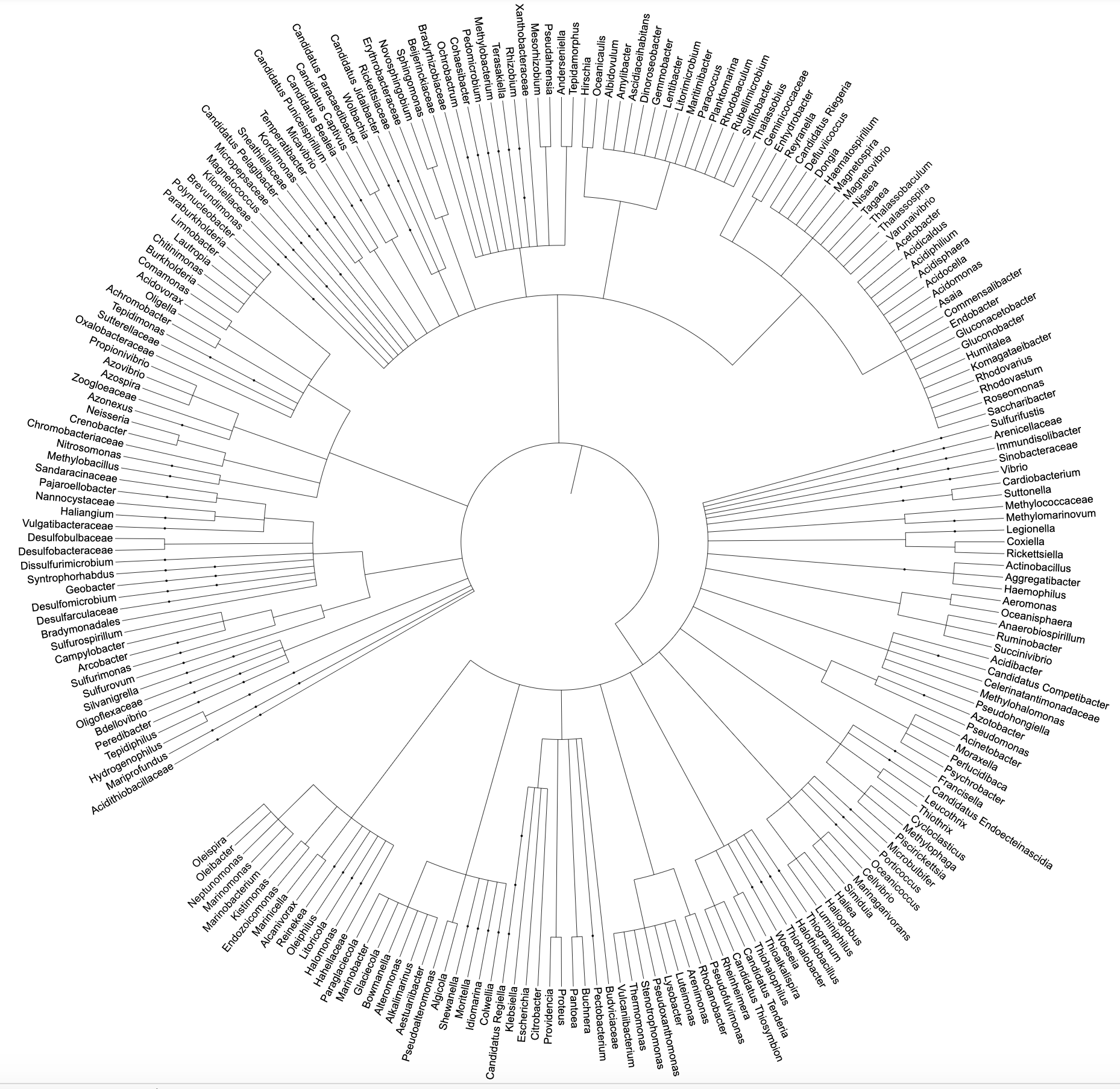


**Figure (S4):** Circular phylogenetic tree presenting fermented juice Proteobacteria at the genus level.


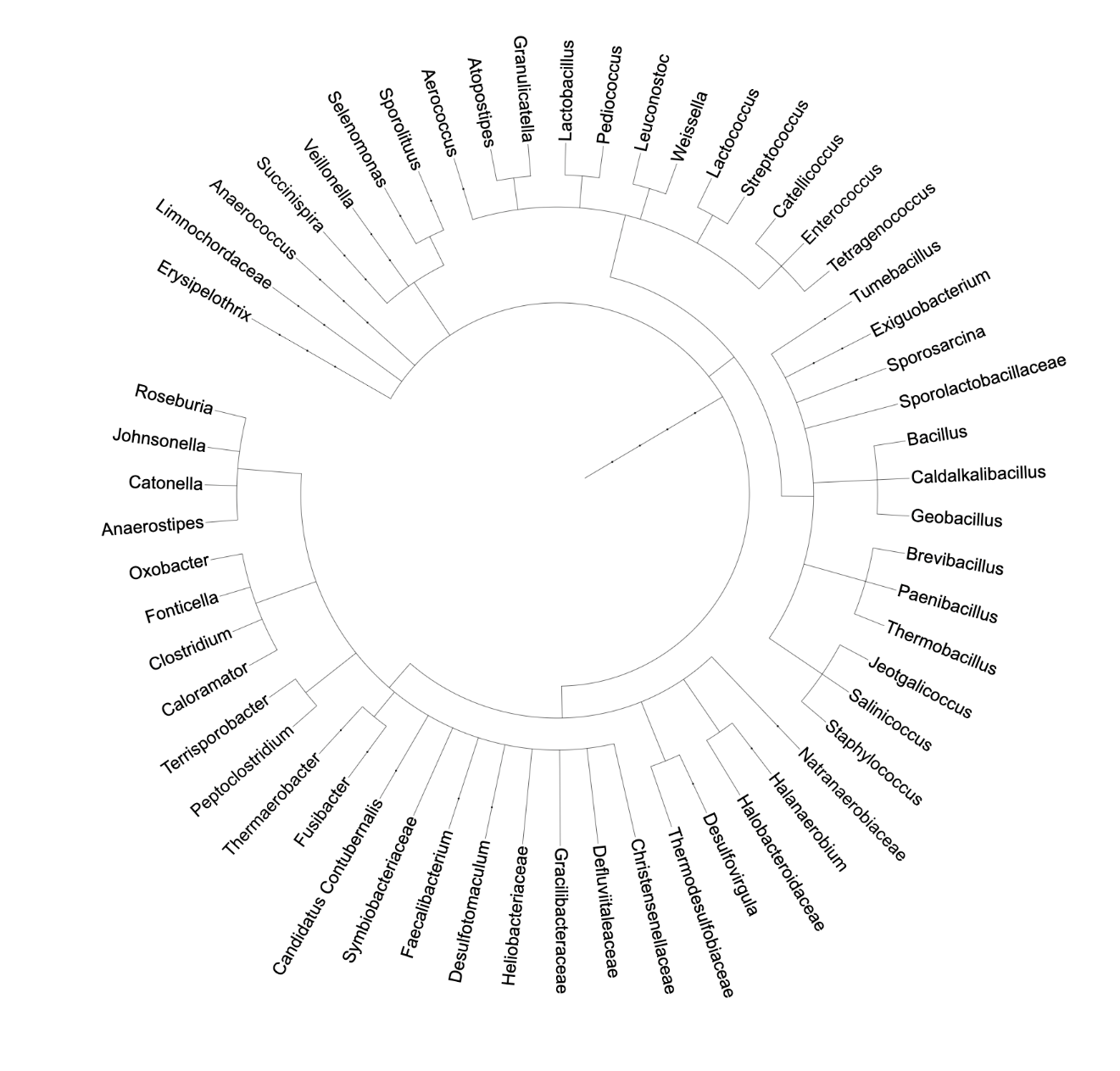


**Figure (S5):** Circular phylogenetic tree presenting fermented juice Firmicutes at the genus level. Arrows refer to the most important genera.


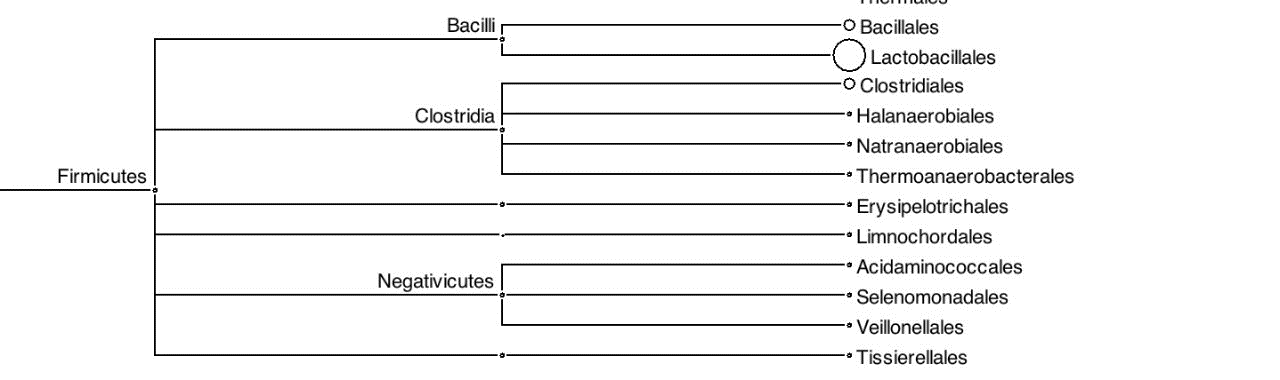


**Figure (S6):** Neighbor-joining phylogenetic diversity of Fimicutes sequences at the order level. The size of *Lactobacillales* circle is relatively larger and it is scaled logarithmically to show the number of reads allocated directly to the taxon.


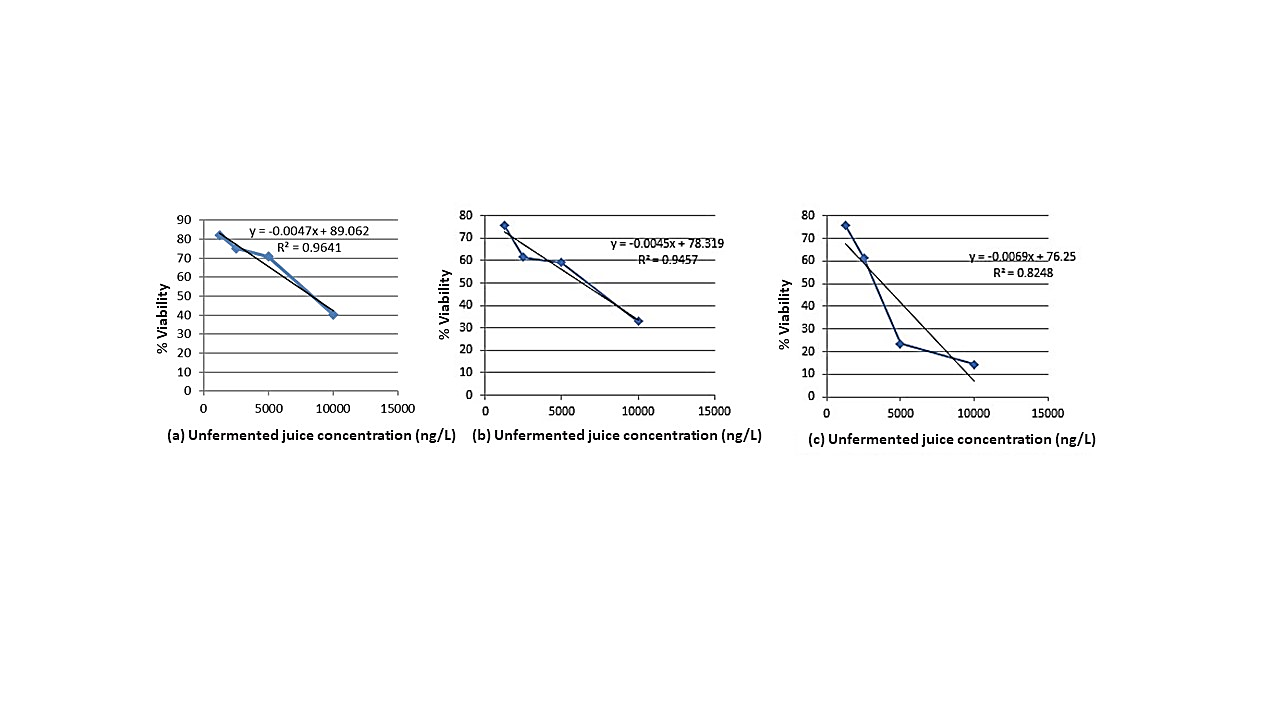


**Figure (S7):** Cytotoxicity assay of unfermented *A. arborescence* juice on (a) Hep-G2 (IC50=8.7 µg/ml), (b) HCT-116 (IC50=6.5 µg/ml), and (c) normal PMNCs (IC50=3.7 µg/ml), using MTT assay.


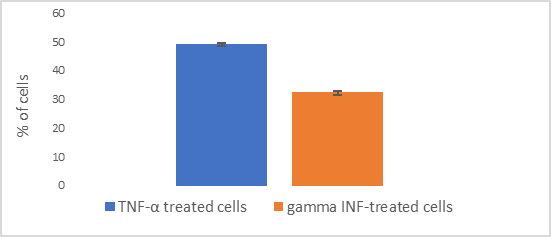

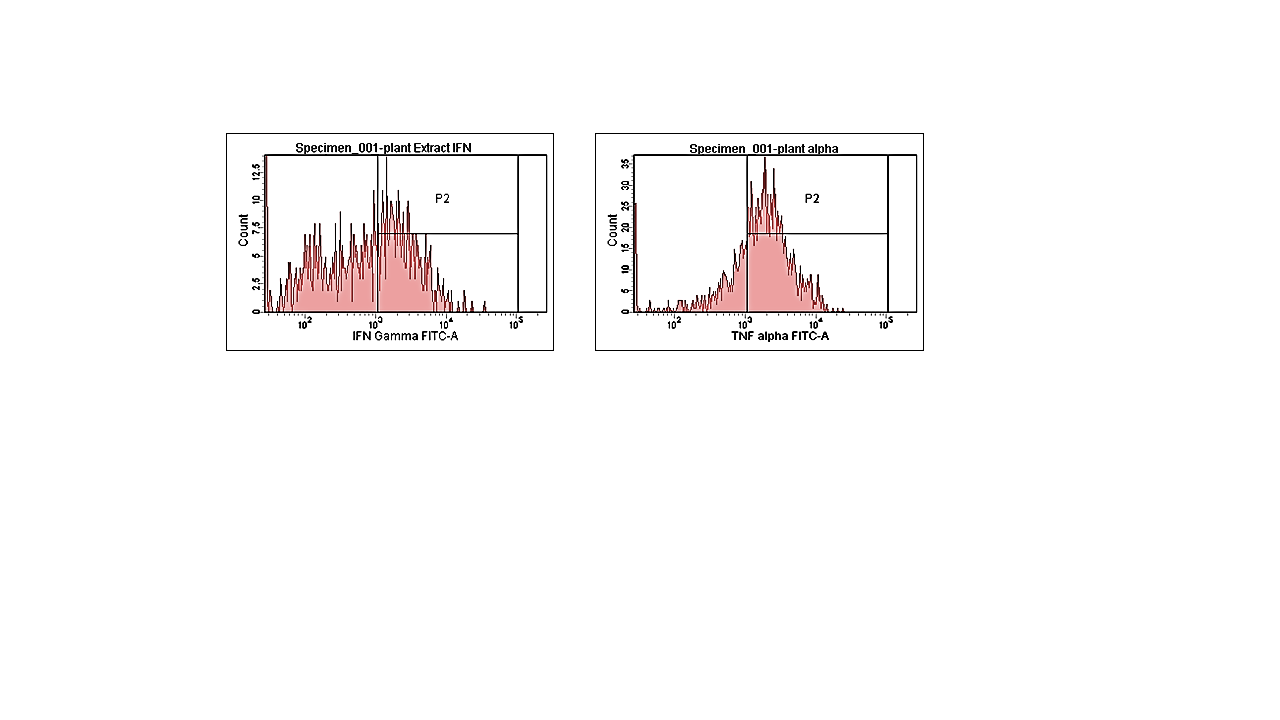


(a)

(b)

**Figure (S8):** Flow cytometric analysis showing the effects of unfermented *aloe* juice on the TNF-α and IFN-γ production by LPS- induced PBMC cell model. (a) Aloe-treated PBMCs for INF-γ showing non-significant increase, and (b) Aloe-treated PBMCs for TNF-α showing nonsignificant reduction. Data are presented in the graph as mean± SE where statistical significance was tested using one-way ANOVA (**P*<0.05, ** *P*<0.001).

**Table (S1): Assessment of antimicrobial activities of unfermented juice using well-diffusion method and MIC determination by broth microdilution assay.**

| **Test pathogens** | **Inhibition zone diameters** (mm)* | **MIC**  (µg/mL) |
| --- | --- | --- |
| ***S. aureus*** | 15±0.57 | 128 |
| ***B. cereus*** | 16±1.7 | 128 |
| ***E. coli*** | 13±0.57 | 256 |
| ***S. typhi*** | 11±1.5 | 256 |
| ***Sh. flexneri*** | 12±0.57 | 128 |
| ***H. pylori*** | 11±1.15 | 256 |
| ***L. monocytogenes*** | 11±0.57 | 128 |
| ***V. cholera*** | 13±0.57 | 128 |
| ***C. albicans*** | 12±0.57 | 256 |

*Values were presented as mean inhibition zone (mm) ± SD of triplicates.
